# Supplementary material for: Identification and categorisation of relevant outcomes for symptomatic uncomplicated gallstone disease: in-depth analysis to inform the development of a core outcome set
Source: BMJ Open. 2021 Jun 24;11(6):e045568. doi: 10.1136/bmjopen-2020-045568 (PMC8231013; doi:10.1136/bmjopen-2020-045568)
Supplement: Supplementary data [file bmjopen-2020-045568supp001.pdf]

APPENDIX 1

UPDATED SEARCH STRATEGY TO IDENTIFY CLINICAL EFFECTIVENESS STUDIES

Ovid MEDLINE(R) Epub Ahead of Print, In-Process & Other Non-Indexed Citations, Ovid MEDLINE(R) Daily and Ovid MEDLINE(R) **1946 – 3rd May 2016** Embase **1980 to 2016 Week 18**

Date of Search: 3rd May 2016

1 cholecystitis/
2 cholecystitis, acute/
3 cholecystolithiasis/
4 gallstones/
5 cholelithiasis/
6 biliary colic/
7 (gall?bladder adj3 (empyema or inflam$)).tw.
8 (biliary colic or gall?stone$ or cholecystitis or cholecystolithiasis).tw.
9 ((pain or biliary symptom$) adj5 (cholecystitis or cholecystolithiasis or gall?bladder)).tw.
10 or/1-9
11 exp Cholecystectomy/
12 cholecystectom$.tw.
13 ((excis$ or remov$) adj4 gall?bladder).tw.
14 ((surgery or surgical) adj5 (cholecystitis or cholecystolithiasis or gall?bladder)).tw.
15 or/11-14
16 exp clinical trial/
17 randomized controlled trial.pt.
18 controlled clinical trial.pt
19 randomi?ed.ab.
20 randomly.ab.
21 trial.ab.
22 placebo.ab.
23 drug therapy.fs.
24 groups.ab.
25 comparative study/
26 (prospective$ or retrospective$).tw.
27 (compare$ or compara$).ti,ab.
28 or/16-27
29 10 and 15 and 28
30 (review or editorial or case report$ or letter).pt.

31 29 not 30

32 limit 31 to human

SEARCH STRATEGY TO IDENTIFY PROMS IN CHOLECYSTITIS

Ovid MEDLINE(R) Epub Ahead of Print, In-Process & Other Non-Indexed Citations, Ovid MEDLINE(R) Daily and Ovid MEDLINE(R) **1946 – 3rd May 2016** Embase **1980 to 2016 Week 18**

Date of Search: 3rd May 2016

1. exp cholecystitis/
2. cholecystolithiasis/
3. gallstones/
4. biliary colic/
5. (gall?bladder adj3 (empyema or inflam$)).tw,kw.
6. (biliary colic or gall?stone$ or cholecystitis or cholecystolithiasis).tw,kw.
7. ((pain or biliary symptom$) adj5 (cholecystitis or cholecystolithiasis or gall?bladder)).tw,kw.
8. or/1-7
9. (core adj3 outcome?).tw,kw.
10. (patient reported adj3 outcome?).tw,kw.
11. prom.tw,kw.
12. 8 and (9 or 10 or 11)
13. *outcome assessment/
14. *"Outcome Assessment (Health Care)"/
15. 8 and (13 or 14)
16. 12 or 15

SEARCH STRATEGY TO IDENTIFY QUALITATIVE STUDIES

Epub Ahead of Print, In-Process & Other Non-Indexed Citations, Ovid MEDLINE(R) Daily and Ovid MEDLINE(R) <1946 to 2nd May 2016

Date of Search: 18th of August, 2016

1. exp cholecystitis/
2. cholecystolithiasis/
3. gallstones/
4. exp Cholecystectomy/

5. biliary colic/
6. (gall?bladder adj3 (empyema or inflam$)).tw,kw.
7. (biliary colic or gall?stone$ or cholecystitis or cholecystolithiasis).tw,kw.
8. ((pain or biliary symptom$) adj5 (cholecystitis or cholecystolithiasis or gall?bladder)).tw,kw.
9. or/1-8
10. qualitative research/
11. exp interviews as topic/
12. focus groups/
13. grounded theory/
14. (qualitative or interview$ or focus group?).tw,kw.
15. (ethno$ or grounded or thematic or interpretive or narrative or discourse analysis or discursive or mixed method$).tw,kw.
16. or/10-15
17. 9 and 16
18. exp animals/ not human/
19. 17 not 18

Appendix 2 . Demographics of participants included in the primary research

**Research type and number of participants** (n)	**Gender**	**Approached to take part in the CGALL trial**<br>Yes/No and number (n)	**Trial consenters (allocated to receive surgery or medical management) or decliners**
Interviews<br>(n=6)	Female=6	Yes (n=6)	Allocated surgery=4<br>Allocated medical management=1<br>Trial non-consenter=1
Audio-consultations<br>(n=20)	Female= 16<br>Male=4	Yes (n=20)	Allocated surgery= 4<br>Allocated medical Management=3<br>Trial non-consenter=13
Focus Groups<br>(n=5)	Female=4<br>Male=1	No	Not Applicable
