# Supplementary material for: Identification and categorisation of relevant outcomes for symptomatic uncomplicated gallstone disease: in-depth analysis to inform the development of a core outcome set
Source: BMJ Open. 2021 Jun 24;11(6):e045568. doi: 10.1136/bmjopen-2020-045568 (PMC8231013; doi:10.1136/bmjopen-2020-045568)
Supplement: Author's manuscript [file bmjopen-2020-045568.draft_revisions.pdf]

BMJ Open

BMJ Open is committed to open peer review. As part of this commitment we make the peer review history of every article we publish publicly available.

When an article is published we post the peer reviewers' comments and the authors' responses online. We also post the versions of the paper that were used during peer review. These are the versions that the peer review comments apply to.

The versions of the paper that follow are the versions that were submitted during the peer review process. They are not the versions of record or the final published versions. They should not be cited or distributed as the published version of this manuscript.

BMJ Open is an open access journal and the full, final, typeset and author-corrected version of record of the manuscript is available on our site with no access controls, subscription charges or pay-per-view fees (http://bmjopen.bmj.com).

If you have any questions on BMJ Open's open peer review process please email info.bmjopen@bmj.com

BMJ Open

Identification and categorisation of relevant outcomes for symptomatic uncomplicated gallstone disease: in depth analysis to inform the development of a core outcome set

Journal:	*BMJ Open*
Manuscript ID	bmjopen-2020-045568
Article Type:	Original research
Date Submitted by the Author:	06-Oct-2020

Complete List of Authors:	Cruickshank, Moira; University of Aberdeen, Health Services Research Unit<br>Newlands, Rumana; University of Aberdeen, Health Services Research Unit<br>Blazeby, Jane; University of Bristol, Department of Social Medicine<br>Ahmed, Irfan; NHS Grampian,<br>Bekheit, Mohamed; NHS Grampian; Department of Surgery, ElKabbary Hospital<br>Brazzelli, Miriam; University of Aberdeen, Health Services Research Unit<br>Croal, Bernard; NHS Grampian, Clinical Biochemistry<br>Innes, Karen; University of Aberdeen, Health Services Research Unit<br>Ramsay, Craig; University of Aberdeen, Health Services Research Unit<br>Gillies, Katie; University of Aberdeen, Health Services Research Unit

Keywords:	Adult gastroenterology < GASTROENTEROLOGY, Gastroenterology < INTERNAL MEDICINE, SURGERY

SCHOLARONE™
Manuscripts

I, the Submitting Author has the right to grant and does grant on behalf of all authors of the Work (as defined in the below author licence), an exclusive licence and/or a non-exclusive licence for contributions from authors who are: i) UK Crown employees; ii) where BMJ has agreed a CC-BY licence shall apply, and/or iii) in accordance with the terms applicable for US Federal Government officers or employees acting as part of their official duties; on a worldwide, perpetual, irrevocable, royalty-free basis to BMJ Publishing Group Ltd ("BMJ") its licensees and where the relevant Journal is co-owned by BMJ to the co-owners of the Journal, to publish the Work in this journal and any other BMJ products and to exploit all rights, as set out in our licence.

The Submitting Author accepts and understands that any supply made under these terms is made by BMJ to the Submitting Author unless you are acting as an employee on behalf of your employer or a postgraduate student of an affiliated institution which is paying any applicable article publishing charge ("APC") for Open Access articles. Where the Submitting Author wishes to make the Work available on an Open Access basis (and intends to pay the relevant APC), the terms of reuse of such Open Access shall be governed by a Creative Commons licence – details of these licences and which Creative Commons licence will apply to this Work are set out in our licence referred to above.

Other than as permitted in any relevant BMJ Author's Self Archiving Policies, I confirm this Work has not been accepted for publication elsewhere, is not being considered for publication elsewhere and does not duplicate material already published. I confirm all authors consent to publication of this Work and authorise the granting of this licence.

For peer review only

Identification and categorisation of relevant outcomes for symptomatic uncomplicated gallstone disease: in depth analysis to inform the development of a core outcome set

Cruickshank M [1,†], Newlands R [1,†], Blazeby JM [2], Ahmed I [1, 3], Bekheit M [3,4], Brazzelli M [1], Croal B [1], Innes K [1], Ramsay C [1], Gillies K [1,*]

[†]Equal contributions

[*]Corresponding author: k.gillies@abdn.ac.uk

1. Health Services Research Unit, Health Sciences Building, Foresterhill, Aberdeen, UK
2. Centre for Surgical Research, University of Bristol, Bristol, UK
3. Department of Surgery, Aberdeen Royal Infirmary, NHS Grampian, Aberdeen, UK.
4. Department of Surgery, ElKabbary Hospital, Alexandria, Egypt

For peer review only

Abstract (298 words)

Background: Many completed trials of interventions for uncomplicated gallstone disease are not as helpful as they could be due to lack of standardization across studies, outcome definition, collection and reporting. This heterogeneity of outcomes across studies hampers useful synthesis of primary studies and ultimately negatively impacts on decision making by all stakeholders. Core outcome sets offer a potential solution to this problem of heterogeneity and concerns over whether the 'right' outcomes are being measured. One of the first steps in core outcome set generation is to identify the range of outcomes reported (in the literature or by patients directly) that are considered important.

Objectives: To develop a systematic map that examines the variation in outcome reporting of interventions for uncomplicated symptomatic gallstone disease, and to identify other outcomes of importance to patients not previously measured or reported in interventional studies.

Results: The literature search identified 794 potentially relevant titles and abstracts of which 137 were deemed eligible for inclusion. A total of 129 RCTs, four gallstone disease specific PROMs, and eight qualitative studies were included. This was supplemented with data from six individual interviews, one focus group (n=5 participants) and analysis of 20 consultations. A total of 386 individual recorded outcomes were identified across the combined evidence: 330 outcomes (which were reported 1147 times) from trials evaluating interventions, 22 outcomes from PROMs, 17 outcomes from existing qualitative studies, and 17 outcomes from primary qualitative research. Areas of overlap between the evidence sources existed but also the primary research contributed new, unreported in this context, outcomes.

Conclusions: This study took a rigorous approach to catalogue and map the outcomes of importance in gallstone disease to enhance the development of the COS 'long' list. A COS for uncomplicated gallstone disease that considers the views of all relevant stakeholders is needed.

Strengths and limitations

- This outcome map review is the first to describe the heterogeneity in outcome reporting within uncomplicated symptomatic gallstone disease.
- There is a detailed analysis of all reported outcomes from a range of study designs (both primary and secondary) reporting outcomes of clinical and/or patient relevance.
- A mixed-methods approach was used in both collection and analysis of data.
- Only studies reported in English-language were included in the analysis
- Quality assessment of included studies was not conducted as the main purpose of the review was to extract clinical outcomes not to assess intervention effectiveness.

For peer review only

Background

Gallstone disease (cholelithiasis) is one of the most common gastrointestinal disorders worldwide The prevalence of gallstones is approximately 10-15% in adult populations and they are more common in women and people over the age of 40.[1] Approximately 80% of those affected by gallstone disease are asymptomatic and can remain so for many years without requiring treatment. However, around 20% of patients with gallstones become symptomatic and develop gallstone related complications. These patients are offered symptom control and/or surgical/endoscopic intervention.[2] A significant number of these patients remain symptomatic only (i.e. experiencing pain) without developing gallstone related complications.[2]

Recommendations from the recent NICE guideline on Gallstone Disease have clearly demonstrated insufficient information for patients on the effect of cholecystectomy on patient outcomes.[3] The Guideline recommends "research is needed to establish the long-term patient benefits and harms, so that appropriate information can be provided to patients to aid decision-making and long-term management of their condition."[3] However, many completed trials are not as helpful as they could be due to lack of standardization across studies, outcome definition, collection and reporting. This heterogeneity of outcomes across studies also hampers useful synthesis of primary studies in meta-analyses and ultimately negatively impacts on decision making by all stakeholders. In addition to the heterogeneity of outcomes currently reported and the problems this causes, measuring the wrong outcomes (i.e. those that are not valued by clinicians or, more importantly, patients) could also be a real risk for many studies if stakeholders are not consulted during the trial design process. One way that these problems with heterogeneity and relevance to stakeholders can be addressed is through the development and use of core outcome sets.[4,5] There is currently no agreed published core outcome set for symptomatic uncomplicated gallstone disease.

Core outcome sets aim to define a minimum set of outcomes that should be considered essential for the evaluation and reporting of specific interventions or conditions (i.e. the set of outcomes that should always be considered and ideally measured in any evaluation).[4,5] There is a growing body of literature to provide support for development of core outcome sets.[5] Specifically, they are developed using consensus methods involving stakeholder groups, such as health professionals and patients, so as to ensure that the outcomes being defined are both clinically and personally relevant for the individuals involved.[4,5] Assessment of a core outcome set is not expected to be mutually exclusive to the measurement of other outcomes. However, a core set will foster greater consistency in outcome reporting between studies and lead to more meaningful data being available to contribute to meta-analysis.[4,5] Moreover, core outcome sets can minimise the threat of outcome reporting bias by

For peer review only

ensuring consistency between what is measured and what is reported.[4,5] Ultimately, they should improve the overall efficiency and quality of the evidence on which health care decisions can be made. A core outcome set for uncomplicated gallstone disease is currently being developed. Details of this project have been registered and included in the Core Outcome Measures in Effectiveness Trials (COMET) Initiative database.[6] Outcome mapping is an important step in the development of core outcome sets, to present and catalogue the outcomes reported to date, and links the literature review and the subsequent process of consultation and consensus.[7] Therefore, the objectives of this paper are to document the outcome mapping process in the development of the core outcome set for symptomatic uncomplicated gallstone disease.

Methods

The protocol for development of the COS is available on the COMET website: http://www.comet-initiative.org/studies/details/927?result=true.

Identification of outcomes relevant for symptomatic uncomplicated gallstone disease.

The identification of outcomes was informed by two sources: existing evidence and new primary research. The specifics of these are detailed below.

1. Identification of outcomes from existing literature:
    a. Outcomes reported in trials of interventions for symptomatic uncomplicated gallstone disease.
    b. Content analysis of individual items within disease specific Patient Reported Outcome Measures (PROMs).
    c. Outcomes from exploratory studies reported by patients with a lived experience of symptomatic uncomplicated gallstone disease.
2. Identification of outcomes of relevance to patients from new primary research.
    a. Interviews and focus groups with patients with a diagnosis of symptomatic uncomplicated gallstone disease and a range of treatments planned or received.
    b. Audio-recordings of consultations for a clinical trial evaluating treatments for symptomatic uncomplicated gallstone disease.

Methods for each of these outcome identification stages are specified below.

Identification of outcomes from existing literature: outcomes reported in trials of interventions for symptomatic uncomplicated gallstone disease and identification of disease specific PROMs.

Reported outcomes of interventions for symptomatic uncomplicated gallstone disease were identified by updating the search strategy for a recent systematic review (Brazzelli et al 2015[8]; this review included two RCTs), by conducting a search for relevant PROMs and by screening the reference lists

For peer review only

of relevant Cochrane reviews. In addition, reference lists of systematic reviews identified by the search strategy were checked for relevant RCTs.

Search methods for identification of studies

Extensive electronic searches were undertaken to identify trials for a project on the clinical effectiveness of cholecystectomy and these are reported in full elsewhere.[8] The databases searched included MEDLINE (1946 to week 37 2012), MEDLINE-in-process (10 September 2012), Embase (1974 to 2012 September 10), Science Citation Index (1970 to 12 September 2012), BIOSIS (1956 to 12 September 2012) and the Cochrane Central Register of Controlled Trials (Issue 9 of 12, 2012). Studies identified from these searches were used to elicit reported outcomes. The MEDLINE and EMBASE searches were updated in May 2016 (Sep 2012 – May 2016) to identify more recent relevant trials.

In addition, a specific search of MEDLINE and EMBASE was undertaken to identify studies that report PROM outcome data for cholecystitis, with records retrieved by the main search for trials excluded to avoid duplication of the results. This search was undertaken in May 2016 (1980-May 2016). The search strategies for MEDLINE and EMBASE are reported in Appendix 1. Inclusion criteria for eligible studies were as follows; Participants: Adults aged over 18 years with symptomatic uncomplicated gallstone disease. Intervention and comparator: Any intervention (surgical or non-surgical management, i.e. expectant management or dietary advice, or medical therapy) used to manage symptomatic uncomplicated gallstone disease in adults. Outcomes: All reported outcomes well eligible for inclusion. Excluded studies included those focusing on asymptomatic gallstone disease or on acute severe cholecystitis, cholangitis, or pancreatitis were not considered suitable for inclusion. In addition, studies including 'complex' gallstone cases i.e. empyema, ascending cholangitis and gallstone ileus, were excluded. Reports published in non-English languages for which a translation could not be organised were also excluded. In addition, lists of included and excluded studies reported in several relevant Cochrane reviews were checked by one reviewer (MC) for potentially relevant studies .[9-14]

Study selection and data extraction

One reviewer (MC) screened all titles and abstracts identified by the two search strategies and a second reviewer (RN) checked a 10% random sample. All full text papers considered potentially eligible were screened by one reviewer (MC) and checked by a second reviewer (RN). One reviewer (MC) extracted details of all outcomes reported (verbatim) and any definition of outcomes provided by the authors. Data was recorded in a Microsoft Excel file. A 10% sample was checked by a second reviewer (RN). Other relevant data (i.e., study and participant characteristics) were extracted by one reviewer (MBe) and checked by a second reviewer (MC). At all stages, disagreement between reviewers was resolved by discussion.

For peer review only

Data extraction from PROMs

From the list of outcomes reported in trials of interventions for symptomatic uncomplicated gallstone disease described above, disease specific PROMS were identified and supplemented with the studies identified in the search. Data were extracted by one reviewer (KG) who recorded the name of the PROM(s), the reported PRO scales and individual verbatim items. The individual verbatim items from each PROM were analysed using an inductive content analysis approach and informed by previous PROM coding work .[15] All PROM items were systematically categorised into conceptual health domains according to the aspect which they aim to capture. Health domains were generated inductively from the identified individual items. Domain mapping was conducted two authors (KG and JB) independently with any conflicts resolved through discussion.

***Identification of outcomes from existing literature*: *outcomes reported as important by patients with a lived experience of uncomplicated gallstone disease*.**

Search methods for identification of studies

A search for relevant qualitative studies was undertaken in August 2016 in the Ovid versions of MEDLINE (from 1966-2016). The search strategies combined search terms for cholecystitis, cholecystectomy, with terms for qualitative research (Appendix 1). Inclusion criteria included (i) studies that have explored (using observations, interviews, focus groups and other qualitative methods) participants' lived experiences of gallstones with specific reference to outcomes of importance. Exclusion criteria included (i) studies that have not used qualitative methods; (ii) any review articles, conference abstracts and those with no full-text articles published or non-English language articles.

Study selection and data extraction

One researcher (RN) screened all abstracts and another (KG) screening a random 10% sample . Full-text articles were obtained for those that were potentially relevant. Two researchers (RN and KG) reviewed all potentially relevant articles to ensure they met the inclusion criteria. To identify additional relevant studies, the reference lists of the included studies were also examined.

Data on study characteristics such as author, publication date, country, focus of investigation, data collection methods, number of participants, and details on sample size were extracted. Additionally, two authors (KG and RN) independently extracted data from two main sources reporting study findings: 1. Direct quotes from participants and; 2. Authors interpretations of participants quotes. This data was recorded verbatim and analysed to identify 'descriptive' thematic codes. Constant comparison method was used to compare findings across studies and an inductive thematic synthesis

For peer review only

was undertaken to generate a list of themes and sub-themes (focused on outcomes) from the data to map across the pre- and post-surgery timeline.[16,17] Throughout this process, the description and wording of the themes were continually revised, and notes made as to how themes and/or sub-themes related and how some could be merged. These findings were discussed further with the research team to finalise the themes across the studies and these were considered, where appropriate, as domains relevant for inclusion in the development of the COS.

Identification of outcomes of relevance to patients from new primary research.

In addition to outcomes reported in existing literature, we conducted primary qualitative research to further inform the identification of outcomes of relevance to patients. Three activities were conducted:

1. Interviews with patients with a diagnosis of symptomatic uncomplicated gallstone disease;
2. A focus group with patients who had undergone cholecystectomy;
3. Analysis of audio-consultations from a trial comparing surgical versus medical management of symptomatic uncomplicated gallstone disease .

Participant identification and invitation

Potential participants for the interviews were identified from the ongoing CGALL trial and were provided with a study participant information leaflet (PIL) either in the clinic or posted to the participant if a decision about CGALL trial entry was made later.[18] The PIL contained a detachable reply-slip to complete and return to the researcher (in a reply paid envelope) if they would like to discuss participating in the interview study. Patients being approached to participate in the CGALL trial were asked (for trial purposes) if they would consent to their consultation being audio-recorded. If consent was obtained, these audio-recordings were then analysed for the identification of outcomes.

Focus group participants were identified through the Scottish Health Research register (SHARE - https://www.registerforshare.org/) and sent an invitation letter asking them to contact the research team if interested in participating. Following initial contact, a researcher phoned the interested participants and ensured they were clear about what the study entailed and arranged a suitable time for the focus group.

Data collection, management and analysis

One author (RN) conducted the interviews over the telephone between [April - August, 2017] . The focus group was conducted by two members of the trial team (KG and the PPI partner BC) on 20th July, 2017. Trial consultations were conducted as standard and four sites across the trial were sampled to inform outcome identification. Informed (written and recorded) consent was obtained from all

For peer review only

participants prior to data collection, and confidentiality of the participants was assured. Participants were encouraged to consider what aspects of their disease or treatment impacted them most, both in terms of physical and social functioning and what improvements they would wish to see in terms of outcomes. Interviews, focus group and audio-consultations were audio-recorded and transcribed verbatim using a professional transcription service. All transcripts were imported into NVivo (Version 10, 2013: QSR International) and analysed using conventional content analysis (i.e. coding categories are derived directly from the text data and are used to interpret meaning from the content).[19] Various themes and sub-themes were generated by one researcher (RN) based on the contents of the transcripts to identify the outcomes stated by the participants, and these were then further discussed (with KG) to finalise the list of outcomes identified across the primary qualitative data. The analysis was oriented to address the aim of identifying the range of outcomes that might be considered important and the reasons used to justify assessment of them as important.

Categorisation of identified outcomes into outcome domains

The list of potential outcomes generated from the systematic evidence search and primary qualitative research formed the basis of a 'long' list of outcomes used to refine the items into a final 'short' list for inclusion in the Delphi stage of core outcome set development. Outcomes were first grouped and reduced according to original source, i.e. the initial long list from the evidence review was reduced for duplication by two members of the research team (MC and IA). A similar process was conducted to de-duplicate the outcomes identified from the PROM coding, qualitative evidence synthesis and primary qualitative research (KG and RN). These outcomes lists were then merged to identify areas of overlap and reduce for further duplication through iterative group discussions (further addressing duplications and relevance of outcomes for effectiveness trials) to produce a final short list OF individual outcomes of relevance in this context.

Individual outcome items were further grouped into broader concept level headings to categorise outcome domains. These concept level headings were informed by other outcome categorisation work in the area of COS and supplemented through study management group discussion. The categorization was performed by one member of the team (KG) and refined through iterative discussions.[15,20,21]

Ethical approval

The primary qualitative research in this study was approved as part of the CGALL trial by the North of Scotland Research Ethics Service [16/NS/0053] and NHS Grampian Research and Development.

Patient and Public Involvement

For peer review only

The outcome mapping work reported in this manuscript has involved the input of patients from inception, through design, conduct and reporting. Co-author BC is a patient partner and has been involved in all phases of project design and delivery and specifically helped to facilitate the patient focus groups to identify outcomes of importance to patients.

Results

Studies identified from the search of existing literature

The literature search identified 794 (633 from the search of trials, 60 from the PROM search and 101 from the search of qualitative literature) potentially relevant titles and abstracts for screening. Of these, 137 (129 from trials and PROMs and 8 qualitative studies) were deemed eligible for inclusion (Figure 1). A total of 137 publications from 129 RCTs, four gallstone disease specific PROMs, and eight qualitative studies were therefore included

Sample characteristics of existing literature and new primary research

Existing literature

Seven studies were conducted in the UK. A further 44 were conducted in countries in Europe, other than the UK, 12 in the USA and 66 in a range of other countries. A total of over 10000 participants were included in the studies, predominantly women (sex ratio around 2.5:1) in their mid 40s (40-53, median 46.1). Studies were mainly single-centre (n=113) and ranged in size from small (14 participants) to large (618 participants) trials (median n=75). A vast majority of trials involved one type of surgery versus another type of surgery, with single incision laparoscopic cholecystectomy versus conventional laparoscopic cholecystectomy being the most common trial configuration.[22-71] Other common configurations were early laparoscopic cholecystectomy versus delayed laparoscopic cholecystectomy,[72-87] mini laparoscopic cholecystectomy versus laparoscopic cholecystectomy,[88-97] mini laparotomy versus laparoscopic cholecystectomy,[98-103] and day-case laparoscopic cholecystectomy versus overnight stay laparoscopic cholecystectomy.[105-107] There were few non-surgical interventions; one study compared shock wave lithotripsy and laparoscopic cholecystectomy,[108] whilst several compared observation with cholecystectomy.[109-110] The four disease specific PROMS for uncomplicated gallstone disease were developed in Canada (n=2), .New Zealand (n=1) and Germany (n=1).[111-114] Two of the PROMS focused on gallstone disease,[111,113] one on gastrointestinal diseases,[114] and the other on quality of life after abdominal surgery.[112] All of the PROMs reported to measure multi-dimensional constructs e.g. quality of life. The PROMs varied in the number of constructs they aimed to assess (ranging from 4-8) and the number of items they asked participants to report on (ranging from 5-41, median = 27).

For peer review only - http://bmjopen.bmj.com/site/about/guidelines.xhtml

The eight qualitative studies from seven different countries: USA (n=2),[115,116] UK,[117] Brazil,[118] Canada,[119] Sweden,[120] the Netherland[121] and Spain[122] were identified including 324 participants (ranged from 12-162, median = 19.5). They were predominantly women and their age ranged from 19-81 years. Seven studies used interviews either face-to-face (n= 5) or by telephone (n=2) and one used focus groups for data collection. All of the treatments being explored in the studies were surgery but the types of surgery varied. Five studies investigated patients' experiences after surgery, two investigated experiences of cholecystitis (i.e. inflammation of the gall bladder) and surgery and another investigated experiences of cholelithiasis (i.e. presence of gallstones). [115-122] Table 1 provides a summary of the characteristics of participants included in the quantitative and qualitative literature review.

Primary research

Six individual interviews, one focus group (n=5 participants) and analysis of 20 consultations (5 each from 4 different hospital sites) were conducted providing data from a total sample of 31 patients. A brief description of the participants is provided in Appendix 2. They included 26 women and five men, 26 of whom had been approached to take part in the CGALL trial (12 trial consenters and 14 trial non-consenters), and 5 patients whom had not been approached about the trial but who had all had a cholecystectomy. The CGALL trial is a randomised controlled trial comparing laparoscopic cholecystectomy with conservative management for preventing recurrent symptoms and complications in adults with uncomplicated symptomatic gallstones.[18]

Outcomes identified from existing literature and new primary research

A total of 386 individual recorded outcomes were identified across the combined evidence from existing literature and new primary research: 330 outcomes (which were reported 1147 times) from trials evaluating interventions, 22 outcomes from PROMs, 17 outcomes from existing qualitative studies, and 17 outcomes from primary qualitative research.

Of the 330 individual outcomes reported in trials, 97 (29.4%) were reported as 'primary outcomes' with only 64 (19.4%) being formally defined and 227 (68.8%) were reported by one study only. The three "verbatim" outcomes which were reported most frequently were: "operating time" (n=103), "postoperative pain" (n=72) and "conversion to open surgery" (n=71). In addition, two studies reported the outcome "conversion". Four studies reported further outcomes relating to "operating time", i.e. "anaesthetic time" (n=1), "surgical time" (n=2), "procedure time" (n=1). Forty studies reported further outcomes relating to pain: "umbilical pain" (n=1), "abdominal pain" (n=9), right upper quadrant pain attacks" (n=3), "chronic post-surgery pain" (n=1), "overall pain" (n=2), "incisional pain" (n=10), "pain in the context of sexual intercourse" (n=1), "visual analogue pain score" (n=2), "pain"

(n=2), “shoulder pain” (n=4), “pain or other symptoms in incision area/port sites” (n=1), “patient reported location of pain at initial postop visit” (n=1), “site of most severe pain” (n=1), “admission due to pain” (n=2), . Table 2 provides a list, and frequency, of all outcomes reported in the included trials.

Some 106 individual items were identified from the four gallstone disease specific PROMS covering 22 individual outcomes, with frequency of each outcome varying across the individual PROMs (Table 3). Included PROMs covered between eight and 14 domains. Pain and emotional outcomes were the most frequently covered with 17 items each (making up 32% of total items) and reported across all four PROMs. There were seven outcomes identified only once (and four of which were identified in one PROM) across the 106 items, which included thirst/dehydration, cognitive, service use, body image, sexual function, regurgitation, swallowing.

Seventeen individual outcomes were identified from the existing qualitative literature (see Table 4). Twelve overlapped with existing outcomes identified in the literature and the PROMS leaving 5 additional outcomes for consideration in the long list, namely: dizziness, fainting, trust, weight, and prevention of additional disease.

The primary qualitative research identified 17 individual outcomes, with the majority (n=14) overlapping with those reported in the previously reviewed evidence (See Table 4). However, three additional outcomes (breathing problems, cough, and mortality) were identified that weren’t in the previous patient focused evidence (PROMs and qualitative literature), with 2 of these (breathing problems, cough) making unique contributions to the overall outcome list.

The 390 individual reported outcomes across the 4 data sources were reduced into a ‘short’ list of outcomes which could be measured in in comparative effectiveness trials (i.e. Phase III pragmatic evections trials) of interventions to treat uncomplicated symptomatic gallstone disease (See Table 5). This resulted in several outcomes being dropped from the long list as deemed not eligible as clinical endpoint outcomes for use in trials of this type (e.g. system and process outcomes such as duration of surgery which might be important in earlier phase trials). This resulted in a list that covered 27 broad outcome domains that contained 41 distinct outcomes. The domains of pain, intra-operative complications, and post-operative complications contained the most outcomes (n=4 each).

Discussion

Currently there is a lack of consistency in the selection, measurement and reporting of outcomes for uncomplicated gallstone disease. This leads to challenges in evidence synthesis and decision-making. A Core Outcome Set would be an important step to improve this situation. This paper which

For peer review only

describes an outcome mapping exercise is the first comprehensive step in the development of a core outcome set. It catalogues and reports the outcomes that have been measured in trials of interventions to treatment uncomplicated gallstone disease. It extends this initial phase of outcome identification to include outcomes from PROMs, published qualitative evidence, and empirical qualitative research. Over 1000 verbatim outcomes were identified and reduced through de-duplication to 390. This was further reduced to 41 outcomes spanning 27 domains. The next steps in this work are to reach consensus for the COS for uncomplicated symptomatic gall stone disease.

As with many other outcome mapping exercises, this first stage of this study highlights the significant heterogeneity that exists within clinical trials comparing treatments for gallstone disease. Of the 334 outcomes, which were reported multiple times across the 129 RCTs, almost 70% were reported by only one study – a finding comparable with other outcome mapping studies.[21,123,124] All of the effort into collection and reporting of these outcomes is likely wasted as it is doubtful that they could be combined with others to make more confident assessment of the effectiveness of treatments. This outcome heterogeneity in existing trials is further emphasised when considering the outcome of pain, which is reported in 72 trials as post-operative pain but also reported in a number of other trials using 15 different outcomes. The four disease specific PROMs identified further extend the problem of outcome heterogeneity. Whilst all of these measures report to capture quality of life, there is variability in both the inclusion and emphasis of domains captured across each of the tools. For example, the most frequently covered domains capture emotion and pain and are reported across all four tools. Fatigue is also reported across all four tools but is only captured by 5 items as opposed to the 17 each for emotion and pain. Nine of the 22 domains are only captured by one of the PROMs, with a further eight domains being captured by 2 PROMs. Again this variability in what individual items are measured in these tools and how they contribute to overall 'quality of life' assessments, raises questions about the legitimacy of combining these measures when evaluating intervention effectiveness. In addition, the relevance of these outcomes to patients must be called into question given the lack of reporting of input from patients in the item inception phase of PROM design across these measures. There are now several reviews of PROMs in other clinical specialties that also provide findings which further highlight the heterogeneity that exists across measures which, on the surface, report to measure similar concepts.[20,125-127]

The different evidence sources contributed to the final outcome short list in a variety of ways with the outcomes reported in previous trials often capturing clinically focussed endpoints and the PROMS and qualitative research providing more patient focused outcomes. When considering what outcomes matter to patients and how this contributes to the outcome mapping in this area, this study used two approaches to ensure patient relevant outcomes were included. The qualitative

For peer review only

evidence synthesis and primary research identified a total of 34 combined (of which 21 were mutually exclusive) outcomes, that were important to patients in terms of their gallstone disease or their perceptions about treatments. Theses outcomes could be broadly grouped into physical and social functioning with most reports from participants focusing on reduction in pain and a desire to 'return to normal'. When compared to the outcome domains reported in the PROMs, there was considerable overlap between the two sources. However, there were some areas of discordance between the different sources, with the qualitative data adding a further 8 outcomes. In addition the qualitative evidence synthesis and the empirical research identified outcomes not previously measured or reported in comparative effectiveness trials for uncomplicated gallstone disease. This value of including evidence from existing literature exploring patients perspective and/or new primary research to identify patient relevant outcomes is gaining traction amongst COS developed.[5] Where most have included outcomes through identification in interviews, the use of qualitative evidence synthesis is growing, and especially in areas where there has previously been a considerable volume of work to draw on.[128] These studies have shown that this work contributes previously unreported outcomes that are of importance to patients and hence underpins the critical nature of this step in COS development. Whether these outcomes identified in these list development stages end up making it into the COS is currently less well evidenced but will be important to know.

Strengths and limitations

This outcome mapping exercise used a systematic search to identify outcomes reported in both quantitative and qualitative studies in the literature. In addition to this rigorous systematic search we supplemented the pool of outcomes already available with new primary qualitative research (through three methods) to further identify outcomes that matter to patients who are experiencing uncomplicates symptomatic gallstone disease. This complementary approach to identification of outcomes has ensured a broad catalogue of both clinically relevant and patient important outcomes. Limitations of this review are linked to the inclusion of only English -language studies and a lack of quality appraisal of included studies, which whilst both of these decisions were fit for purpose for this activity, may have introduced potential reporting and selection bias.

Conclusions

This study took a rigorous approach to catalogue and map the outcomes of importance in gallstone disease to enhance the development of the COS 'long' list. The synthesis of data from the four different evidence sources further underpinned the need for a COS in this space due to the heterogeneity of outcome measurement and reporting. However, the extensive use of data sources to contribute to the development of the list of outcomes for further consensus agreement, did

For peer review only

highlight ‘new’ outcomes that have not been previously reported for trials evaluating interventions for gallstone disease and many of these ‘new’ outcomes were those reported by patients. This comprehensive approach to the development of the long list, and then ultimately the short list for scoring in a COS, gives confidence that both clinically relevant and patient focused outcomes have been considered and have the potential to be represented in the agreed COS.

Acknowledgements

The authors thank the HSRU Information Scientist for help with the database searches and the CGALL trial team and management group for ongoing advice and contribution to the study. We would also like to thank the patients who contributed their time to for the interviews.

(4706 words)

For peer review only

References

1. Barbara L, Sama C, Labate AMM. A population study on the prevalence of gallstone disease: The Sirmione study. *Hepatology* 1987;7:913-7
2. Festi D, Reggiani MLB, Attili AF, Loria P, Pazzi P, Scaioli E, et al. Natural history of gallstone disease: Expectant management or active treatment? Results from a population-based cohort study. *J Gastroenterol Hepatol* 2010;25:719-24
3. Gallstone disease. NICE Clinical Guideline CG188. *National Insititute for Health and Care Excellence* 2014. http://www.nice.org.uk/guidance/cg188 [accessed April 2016]
4. Williamson PR, Altman DG, Blazeby JM, Clarke M, Devane D, Gargon E, Tugwell P. Developing core outcome sets for clinical trials: issues to consider. Trials. 2012 Aug 6;13:132
5. Davis K, Gorst SL, Harman N, Smith V, Gargon E, Altman DG, Blazeby JM, Clarke M, Tunis S, Williamson PR. Choosing important health outcomes for comparative effectiveness research: An updated systematic review and involvement of low and middle income countries. PLoS One. 2018 Feb 13;13(2):e0190695.
6. http://www.comet-initiative.org/studies/details/927?result=true *Last accessed 24/009/20*
7. Kaufman J, Ryan R, Bosch-Capblanch X, Cartier Y, Cliff J, Glenton C, Lewin S, Rada G, Ames H, Muloliwa AM, Oku A, Oyo-Ita A, Hill S. Outcomes mapping study for childhood vaccination communication: too few concepts were measured in too many ways. J Clin Epidemiol. 2016 Apr;72:33-44.
8. Brazzelli M, Cruickshank M, Kilonzo M, Ahmed I, Stewart F, McNamee P, Elders A, Fraser C, Avenell A, Ramsay C. Systematic review of the clinical and cost effectiveness of cholecystectomy versus observation/conservative management for uncomplicated symptomatic gallstones or cholecystitis. Surg Endosc. 2015 Mar;29(3):637-47. doi: 10.1007/s00464-014-3712-6.
9. Gurusamy, K.S., Samraj, K., Fusai, G. and Davidson, B.R., 2008. Early versus delayed laparoscopic cholecystectomy for biliary colic. *Cochrane Database of Systematic Reviews*, (4).
10. Gurusamy, K.S., Koti, R., Fusai, G. and Davidson, B.R., 2013. Early versus delayed laparoscopic cholecystectomy for uncomplicated biliary colic. *Cochrane Database of Systematic Reviews*, (6).
11. Gurusamy, K.S., Davidson, C., Gluud, C. and Davidson, B.R., 2013. Early versus delayed laparoscopic cholecystectomy for people with acute cholecystitis. *Cochrane Database of Systematic Reviews*, (6).
12. Gurusamy, K.S., Samraj, K., Fusai, G. and Davidson, B.R., 2012. Robot assistant versus human or another robot assistant in patients undergoing laparoscopic cholecystectomy. *Cochrane database of systematic reviews*, (9).
13. Gurusamy, K.S., Vaughan, J., Ramamoorthy, R., Fusai, G. and Davidson, B.R., 2013. Miniports versus standard ports for laparoscopic cholecystectomy. *Cochrane Database of Systematic Reviews*, (8).
14. Gurusamy, K.S., Vaughan, J., Rossi, M. and Davidson, B.R., 2014. Fewer-than-four ports versus four ports for laparoscopic cholecystectomy. *Cochrane Database of Systematic Reviews*, (2).
15. Macefield, R.C., Jacobs, M., Korfage, I.J. *et al.* Developing core outcomes sets: methods for identifying and including patient-reported outcomes (PROs). *Trials* **15,** 49 (2014). https://doi.org/10.1186/1745-6215-15-49
16. Hewitt-Taylor J. Use of constant comparative analysis in qualitative research. Nurs Stand. 2001 Jul 4-10;15(42):39-42.
17. Thomas J, Harden A. Methods for the thematic synthesis of qualitative research in systematic reviews. BMC Med Res Methodol. 2008 Jul 10;8:45
18. https://w3.abdn.ac.uk/hsru/C-GALL/Public/Public/index.cshtml *Last accessed 24/009/20*
19. Hsieh HF, Shannon SE. Three approaches to qualitative content analysis. Qual Health Res. 2005 Nov;15(9):1277-88
20. Outcome reporting in bariatric surgery: an in-depth analysis to inform the development of a core outcome set, the BARIACT Study Hopkins, J C; Howes, N; Chalmers, K; Savovic, J; Whale, K;

Coulman, K D; Welbourn, R; Whistance, R N; Andrews, R C; Byrne, J P; Mahon, D; Blazeby, J M Obesity reviews. , 2015, Vol.16(1), p.88-106

21. Coulman KD, Hopkins J, Brookes ST, Chalmers K, Main B, Owen-Smith A, Andrews RC, Byrne J, Donovan JL, Mazza G, Reeves BC, Rogers CA, Thompson JL, Welbourn R, Wordsworth S, Blazeby JM; BARIACT working group. A Core Outcome Set for the Benefits and Adverse Events of Bariatric and Metabolic Surgery: The BARIACT Project. PLoS Med. 2016 Nov 29;13(11):e1002187.
22. Abd Ellatif ME, Askar WA, Abbas AE, Noaman N, Negm A, El-Morsy G, El Nakeeb A, Magdy A, Amin M. Quality-of-life measures after single-access versus conventional laparoscopic cholecystectomy: a prospective randomized study. Surgical endoscopy. 2013 Jun 1;27(6):1896-906.
23. ClinicalTrials.gov. *Safety and Cost-effectiveness Study of Single Port Laparoscopic Cholecystectomies (SPoCOT*). Bethesda, MD: National Library of Medicine; 2009 [https://clinicaltrials.gov/ct2/show/NCT00974194]
24. Aprea G, Bottazzi EC, Guida F, Masone S, Persico G. Laparoendoscopic single site (LESS) versus classic video-laparoscopic cholecystectomy: a randomized prospective study. Journal of Surgical Research. 2011 Apr 1;166(2):e109-12.
25. Artis T, Kucuk C, Akay A, Zararsiz G, Sozuer E. Prospective randomized study comparing single incision vs standard laparoscopic cholecystectomy. Surg Endosc 2014;28:S1-S53.
26. UMIN Clinical Trials Registry. *Randomized controlled trial to evaluate the superiority of the single port cholecystectomy on reduction of postoperative pain, compared with conventional laparoscopic cholecystectomy*. Japan: University Hospital Medical Information Network; 2009 [https:// upload.umin.ac.jp/cgi-open-bin/ctr_e/ctr_view.cgi?recptno=R000003103]
27. ClinicalTrials.gov. *Single port laparoscopic cholecystectomy versus four port laparoscopic cholecystectomy: impact on postoperative pain*. Bethesda, MD: National Library of Medicine; 2011 [https://clinicaltrials.gov/ct2/show/NCT01348620]
28. Bignell M, Lewis MP, Cheong EC, Rhodes M. A prospective, randomized, single-blind trial of 5-mm versus 3-mm ports for laparoscopic cholecystectomy: is smaller better?. Surgical endoscopy. 2013 Oct 1;27(10):3616-21.
29. Bingener J, Skaran P, McConico A, Novotny P, Wettstein P, Sletten DM, Park M, Low P, Sloan J. A double-blinded randomized trial to compare the effectiveness of minimally invasive procedures using patient-reported outcomes. Journal of the American College of Surgeons. 2015 Jul 1;221(1):111-21.
30. ClinicalTrials.gov. *A comparison of single port and four port laparoscopic gallbladder removal CENoMIP*. Bethesda, MD: National Library of Medicine; 2011 [https://clinicaltrials.gov/ct2/show/NCT01489436]
31. Borle FR, Mehra BK, Jain S. Evaluation of pain scores after single-incision and conventional laparoscopic cholecystectomy: A randomized control trial in a rural Indian population. Asian journal of endoscopic surgery. 2014 Jan;7(1):38-42.
32. Brown KM, Moore BT, Sorensen GB, Boettger CH, Tang F, Jones PG, Margolin DJ. Patient-reported outcomes after single-incision versus traditional laparoscopic cholecystectomy: a randomized prospective trial. Surgical endoscopy. 2013 Sep 1;27(9):3108-15.
33. Bucher P, Pugin F, Buchs NC, Ostermann S, Morel P. Randomized clinical trial of laparoendoscopic single-site versus conventional laparoscopic cholecystectomy. British Journal of Surgery. 2011 Dec;98(12):1695-702.
34. Cao ZG, Cai W, Qin MF, Zhao HZ, Yue P, Li Y. Randomized clinical trial of single-incision versus conventional laparoscopic cholecystectomy: short-term operative outcomes. Surgical Laparoscopy Endoscopy & Percutaneous Techniques. 2011 Oct;21(5):311.
35. Chang SK, Wang YL, Shen L, Iyer SG, Shaik AB, Lomanto D. Interim report: A randomized controlled trial comparing postoperative pain in single-incision laparoscopic cholecystectomy and conventional laparoscopic cholecystectomy. Asian journal of endoscopic surgery. 2013 Feb;6(1):14-20.

36. Chang SK, Wang YL, Shen L, Iyer SG, Madhavan K. A randomized controlled trial comparing post-operative pain in single-incision laparoscopic cholecystectomy versus conventional laparoscopic cholecystectomy. World Journal of Surgery. 2015 Apr 1;39(4):897-904.
37. Netherlands trial register. *Single incision, miniport or conventional laparoscopic surgery for uncomplicated symptomatic cholecystolithiasis*. Netherlands: Netherlands Trial Registry; 2011, [https://www.trialregister.nl/trial/2952]
38. Emre TK, Ilhan E, Yakan S, Cengiz F, Senlikci A, Aktürk HE. Single-port laparoscopic cholecystectomy versus the classical four port laparoscopic cholecystectomy: a randomized prospective clinical trial. Minerva chirurgica. 2014 Feb;69(1):1.
39. Herrero EF, Cugat EA, García MD, Camps JL, Porta RC, Carvajal FL, Rodríguez AC. A randomised prospective comparative study between laparoscopic cholecystectomy and single port cholecystectomy in a major outpatient surgery unit. Cirugia espanola. 2012 Dec;90(10):641-6.
40. Guo W, Liu Y, Han W, Liu J, Jin L, Li JS, Zhang ZT. Randomized trial of immediate postoperative pain following single-incision versus traditional laparoscopic cholecystectomy. Chinese medical journal. 2015 Dec 20;128(24):3310.
41. ClinicalTrials.gov. A trial of single port laparoscopic cholecystectomy versus four port laparoscopic cholecystectomy. Bethesda, MD: National Library of Medicine; 2009 [https://clinicaltrials.gov/ct2/show/NCT00892879]
42. Hattori K, Watanabe K, Ogino H, Shimoyana R, Kawachi J, Takeyama H. Prospective comparison between single incision laparoscopic cholecystectomy and needlescopic cholecystectomy. Surgical Endoscopy 2012;26:S249-S430.
43. Hauters P, Auvray S, Cardin JL, Papillon M, Delaby J, Dabrowski A, Framery D, Valverde A, Rubay R, Siriser F, Malvaux P. Comparison between single-incision and conventional laparoscopic cholecystectomy: a prospective trial of the Club Coelio. Surgical endoscopy. 2013 May 1;27(5):1689-94.
44. Jørgensen LN, Rosenberg J, Al-Tayar H, Assaadzadeh S, Helgstrand F, Bisgaard T. Randomized clinical trial of single-versus multi-incision laparoscopic cholecystectomy. British Journal of Surgery. 2014 Mar;101(4):347-55.
45. Khorgami Z, Shoar S, Anbara T, Soroush A, Nasiri S, Movafegh A, Aminian A. A randomized clinical trial comparing 4-port, 3-port, and single-incision laparoscopic cholecystectomy. Journal of investigative surgery. 2014 Jun 1;27(3):147-54.
46. Lai EC, Yang GP, Tang CN, Yih PC, Chan OC, Li MK. Prospective randomized comparative study of single incision laparoscopic cholecystectomy versus conventional four-port laparoscopic cholecystectomy. The American Journal of Surgery. 2011 Sep 1;202(3):254-8.
47. Leung D, Yetasook AK, Carbray J, Butt Z, Hoeger Y, Denham W, Barrera E, Ujiki MB. Single-incision surgery has higher cost with equivalent pain and quality-of-life scores compared with multiple-incision laparoscopic cholecystectomy: a prospective randomized blinded comparison. Journal of the American College of Surgeons. 2012 Nov 1;215(5):702-8.
48. Lirici MM, Califano AD, Angelini P, Corcione F. Laparo-endoscopic single site cholecystectomy versus standard laparoscopic cholecystectomy: results of a pilot randomized trial. The American Journal of Surgery. 2011 Jul 1;202(1):45-52.
49. Luna RA, Nogueira DB, Varela PS, Neto ED, Norton MJ, Luciana do Carmo BR, Peixoto AM, De Mendonça YL, Bendet I, Fiorelli RA, Dolan JP. A prospective, randomized comparison of pain, inflammatory response, and short-term outcomes between single port and laparoscopic cholecystectomy. Surgical endoscopy. 2013 Apr 1;27(4):1254-9.
50. Lurje G, Raptis DA, Steinemann DC, Amygdalos I, Kambakamba P, Petrowsky H, Lesurtel M, Zehnder A, Wyss R, Clavien PA, Breitenstein S. Cosmesis and body image in patients undergoing single-port versus conventional laparoscopic cholecystectomy: a multicenter double-blinded randomized controlled trial (SPOCC-trial). Annals of surgery. 2015 Nov 1;262(5):728-35.

51. Ma J, Cassera MA, Spaun GO, Hammill CW, Hansen PD, Aliabadi-Wahle S. Randomized controlled trial comparing single-port laparoscopic cholecystectomy and four-port laparoscopic cholecystectomy. Annals of surgery. 2011 Jul 1;254(1):22-7.
52. Madureira FA, Manso JE, Madureira Filho D, Iglesias AC. Inflammation in laparoendoscopic single-site surgery versus laparoscopic cholecystectomy. Surgical innovation. 2014 Jun;21(3):263-8.
53. Marks JM, Phillips MS, Tacchino R, Roberts K, Onders R, DeNoto G, Gecelter G, Rubach E, Rivas H, Islam A, Soper N. Single-incision laparoscopic cholecystectomy is associated with improved cosmesis scoring at the cost of significantly higher hernia rates: 1-year results of a prospective randomized, multicenter, single-blinded trial of traditional multiport laparoscopic cholecystectomy vs single-incision laparoscopic cholecystectomy. Journal of the American College of Surgeons. 2013 Jun 1;216(6):1037-47.
54. McGregor CG, Sodergren MH, Aslanyan A, Wright VJ, Purkayastha S, Darzi A, Paraskeva P. Evaluating systemic stress response in single port vs. multi-port laparoscopic cholecystectomy. Journal of Gastrointestinal Surgery. 2011 Apr 1;15(4):614-22.
55. Mehmood ZA, Subhan AN, Ali NA, Rasul SH, Iqbal MA, Khan RA, Karim MT, Shafqutallah SM, Baig MS. Four port versus single incision laparoscopic cholecystectomy. Journal of Surgery Pakistan (International). 2010 Jul;15(3):122.
56. Moran M, Gundogdu E, Dizen H, Akgun AE, Ozmen MM. Single vs two vs three vs four incision laparoscopic cholecystectomy: a randomized controlled trial. Surgical Endoscopy 2011;25:S1-S53.
57. ClinicalTrials.gov. *EndoCone single port versus conventional multi-port laparoscopic approach*. Bethesda, MD: National Library of Medicine; 2012 [https://clinicaltrials.gov/ct2/show/NCT01709877]
58. Ospanov OB, Dildabekov GB. Single-site laparoscopic cholecystectomy using a multiple transumbilical port. Surgical Endoscopy 2012;26:S53-S140.
59. Pan MX, Jiang ZS, Cheng Y, Xu XP, Zhang Z, Qin JS, He GL, Xu TC, Zhou CJ, Liu HY, Gao Y. Single-incision vs three-port laparoscopic cholecystectomy: prospective randomized study. World journal of gastroenterology: WJG. 2013 Jan 21;19(3):394.
60. Pappas-Gogos G, Tellis CC, Trypsianis G, Tsimogiannis KE, Tsimoyiannis EC, Simopoulos CE, Pitiakoudis M, Tselepis AD. Oxidative stress in multi-port and single-port cholecystectomy. journal of surgical research. 2015 Mar 1;194(1):101-6.
61. Pathania BS. A comparative clinical evaluation of single incision laparoscopic cholecystectomy with single and multiport access versus conventional multi-port laparoscopic cholecystectomy. European Surgical Research 2013;50(Suppl 1):1-162.
62. Rašić Ž, Schwarz D, Adam Nesek V, Zoričić I, Sever M, Rašić D, Lojo N. Single incision laparoscopic cholecystectomy–a new advantage of gallbladder surgery. Collegium antropologicum. 2010 Jun 30;34(2):595-8.
63. Sasaki A, Ogawa M, Tono C, Obara S, Hosoi N, Wakabayashi G. Single-port versus multiport laparoscopic cholecystectomy: a prospective randomized clinical trial. Surgical Laparoscopy Endoscopy & Percutaneous Techniques. 2012 Oct 1;22(5):396-9.
64. Sinan H, Demirbas S, Ozer MT, Sucullu I, Akyol M. Single-incision laparoscopic cholecystectomy versus laparoscopic cholecystectomy: a prospective randomized study. Surgical Laparoscopy Endoscopy & Percutaneous Techniques. 2012 Feb 1;22(1):12-6.
65. IRCT.ir. *The comparison between result of single port laparoscopic cholecystectomy with four ports cholecystectomy*. Tehran, Iran: IRCT; 2011 [https://www.irct.ir/trial/6802]
66. Sulu B, Yildiz BD, Ilingi ED, Gunerhan Y, Cakmur H, Anuk T, Yildiz B, Koksal N. Single Port vs. Four Port Cholecystectomy--Randomized Trial on Quality of Life. Advances in clinical and experimental medicine: official organ Wroclaw Medical University. 2015;24(3):469-73.
67. Tono C. Single-port versus multi-port laparoscopic cholecystectomy: a randomised clinical trial. Surgical Endoscopy 2012;26:S53-S140.
68. Ugurlu MU, Oruc MT. Comparison of single-incision laparoscopic cholecystectomy with standard laparoscopic approach. Surgical Endoscopy 2013;27:S304-S503.

69. ClinicalTrials.gov. *A randomised comparison betweennsingle incision laparoscopic cholecystectomy and standard laparoscopic cholecystectomy*. Bethesda, MD: National Library of Medicine; 2010 [https://clinicaltrials.gov/ct2/show/ NCT01094379]
70. Vilallonga R, Barbaros U, Sümer A, Demirel T, Fort JM, González O, Rodriguez N, Carrasco MA. Single-port transumbilical laparoscopic cholecystectomy: a prospective randomised comparison of clinical results of 140 cases. Journal of minimal access surgery. 2012 Jul;8(3):74.
71. Zapf M, Yetasook A, Leung D, Salabat R, Denham W, Barrera E, Butt Z, Carbray J, Du H, Wang CE, Ujiki M. Single-incision results in similar pain and quality of life scores compared with multi-incision laparoscopic cholecystectomy: a blinded prospective randomized trial of 100 patients. Surgery. 2013 Oct 1;154(4):662-71.
72. Agrawal R, Sood KC, Agarwal B. Evaluation of early versus delayed laparoscopic cholecystectomy in acute cholecystitis. Surgery Research and Practice. 2015 Feb 3;2015.
73. Faizi KS, Ahmed I, Ahmad H. Comparison of early versus delayed laparoscopic cholecystectomy: choosing the best. Pak J Medical Health Sci. 2013;7:212-5.
74. Gul R, Dar RA, Sheikh RA, Salroo NA, Matoo AR, Wani SH. Comparison of early and delayed laparoscopic cholecystectomy for acute cholecystitis: experience from a single center. North American journal of medical sciences. 2013 Jul;5(7):414.
75. Gutt CN, Encke J, Köninger J, Harnoss JC, Weigand K, Kipfmüller K, Schunter O, Götze T, Golling MT, Menges M, Klar E. Acute cholecystitis: early versus delayed cholecystectomy, a multicenter randomized trial (ACDC study, NCT00447304). Annals of surgery. 2013 Sep 1;258(3):385-93.
76. Johansson M, Thune A, Blomqvist A, Nelvin L, Lundell L. Management of acute cholecystitis in the laparoscopic era: results of a prospective, randomized clinical trial. Journal of gastrointestinal surgery. 2003 Jul 1;7(5):642-5.
77. Johansson M, Thune A, Nelvin L, Stiernstam M, Westman B, Lundell L. Randomized clinical trial of open versus laparoscopic cholecystectomy in the treatment of acute cholecystitis. British journal of surgery. 2005 Jan 1;92(1):44-9.
78. Kolla SB, Aggarwal S, Kumar A, Kumar R, Chumber S, Parshad R, Seenu V. Early versus delayed laparoscopic cholecystectomy for acute cholecystitis: a prospective randomized trial. Surgical Endoscopy and Other Interventional Techniques. 2004 Sep 1;18(9):1323-7.
79. Lai PB, Kwong KH, Leung KL, Kwok SP, Wchan AC, Chung SC, Lau WY. Randomized trial of early versus delayed laparoscopic cholecystectomy for acute cholecystitis. British journal of surgery. 1998 Jun 1;85(6):764-7.
80. Lo CM, Liu CL, Fan ST, Lai EC, Wong J. Prospective randomized study of early versus delayed laparoscopic cholecystectomy for acute cholecystitis. Annals of surgery. 1998 Apr;227(4):461.
81. Macafee DA, Humes DJ, Bouliotis G, Beckingham IJ, Whynes DK, Lobo DN. Prospective randomized trial using cost–utility analysis of early versus delayed laparoscopic cholecystectomy for acute gallbladder disease. British journal of surgery. 2009 Sep;96(9):1031-40.
82. Mare LD, Saadi A, Roulin D, Demartines N, Halkic N. Delayed versus early laparoscopic cholecystectomy for acute cholecystitis: a prospective randomized study. HBP 2012;14(Suppl 2):107-287.
83. Özkardeş AB, Tokaç M, Dumlu EG, Bozkurt B, Çiftçi AB, Yetişir F, Kılıç M. Early versus delayed laparoscopic cholecystectomy for acute cholecystitis: a prospective, randomized study. International surgery. 2014;99(1):56-61.
84. Saber A, Hokkam EN. Operative outcome and patient satisfaction in early and delayed laparoscopic cholecystectomy for acute cholecystitis. Minimally invasive surgery. 2014 Jan 1;2014.
85. Salman B, Yüksel O, Irkörücü O, Akyürek N, Tezcaner T, Doğan İ, Erdem Ö, Tatlicioğlu E. Urgent laparoscopic cholecystectomy is the best management for biliary colic. Digestive surgery. 2005;22(1-2):95-9.
86. Verma S, Agarwal PN, Bali RS, Singh R, Talwar N. Early versus delayed laparoscopic cholecystectomy for acute cholecystitis: a prospective randomized trial. International Scholarly Research Notices. 2013;2013.

87. Yadav RP, Adhikary S, Agrawal CS, Bhattarai B, Gupta RK, Ghimire A. A comparative study of early vs. delayed laparoscopic cholecystectomy in acute cholecystitis. Kathmandu University medical journal. 2009;7(1):16-20.
88. Alhashemi M, Almahroos M, Fiore JF, Kaneva P, Gutierrez JM, Neville A, Vassiliou MC, Fried GM, Feldman LS. Impact of miniport laparoscopic cholecystectomy versus standard port laparoscopic cholecystectomy on recovery of physical activity: a randomized trial. Surgical endoscopy. 2017 May 1;31(5):2299-309.
89. Alponat A, Çubukçu A, Gönüllü N, Cantürk Z, Öbay O. Is minisite cholecystectomy less traumatic? Prospective randomized study comparing minisite and conventional laparoscopic cholecystectomies. World journal of surgery. 2002 Dec 1;26(12):1437-40.
90. de Carvalho LF, Fierens K, Kint M. Mini-laparoscopic versus conventional laparoscopic cholecystectomy: a randomized controlled trial. Journal of Laparoendoscopic & Advanced Surgical Techniques. 2013 Feb 1;23(2):109-16.
91. Gupta A, Shrivastava UK, Kumar P, Burman D. Minilaparoscopic versus laparoscopic cholecystectomy: a randomised controlled trial. Tropical gastroenterology: official journal of the Digestive Diseases Foundation. 2005 Jul 1;26(3):149-51.
92. Hsieh CH. Early minilaparoscopic cholecystectomy in patients with acute cholecystitis. The American journal of surgery. 2003 Apr 1;185(4):344-8.
93. Huang MT, Wang W, Wei PL, Chen RJ, Lee WJ. Minilaparoscopic and laparoscopic cholecystectomy: a comparative study. Archives of Surgery. 2003 Sep 1;138(9):1017-23.
94. Leggett PL, Churchman-Winn R, Miller G. Minimizing ports to improve laparoscopic cholecystectomy. Surgical endoscopy. 2000 Jan 1;14(1):32-6.
95. Novitsky YW, Kercher KW, Czerniach DR, Kaban GK, Khera S, Gallagher-Dorval KA, Callery MP, Litwin DE, Kelly JJ. Advantages of mini-laparoscopic vs conventional laparoscopic cholecystectomy: results of a prospective randomized trial. Archives of Surgery. 2005 Dec 1;140(12):1178-83.
96. Saad S, Strassel V, Sauerland S. Randomized clinical trial of single-port, minilaparoscopic and conventional laparoscopic cholecystectomy. British Journal of Surgery. 2013 Feb;100(3):339-49.
97. Velázquez-Mendoza JD, Villagrán-Murillo FJ, González-Ojeda A. Minilaparotomy vs. laparoscopic cholecystectomy: results of a randomized clinical trial. Cirugía y Cirujanos. 2012;80(2):115-21.
98. Ahmed W, Gaber A, Elmoghazy W, Elsharkawy K. Mini-laparotomy cholecystectomy versus laparoscopic cholecystectomy: a randomized controlled trial. Surgical Endoscopy 2016;30:S63-S156.
99. Aspinen S, Harju J, Juvonen P, Kokki H, Remes V, Scheinin T, Eskelinen M. A prospective, randomized multicenter study comparing conventional laparoscopic cholecystectomy versus minilaparotomy cholecystectomy with ultrasonic dissection as day surgery procedure—1-year outcome. Scandinavian journal of gastroenterology. 2014 Nov 1;49(11):1336-42.
100. Aspinen A, Harju J, Juvonen P, Kokki H, Remes V,Scheinin, Eskelinen M. Minilaparotomy cholecystectomy with ultrasonic dissection versus conventional laparoscopic cholecystectomy-1 year outcome. Surgical Endoscopy 2015;29:S75-S252.
101. Harju J. Comparison of the quality of life after minilaparotomy cholecystectomy versus laparoscopic cholecystectomy: a prospective randomized study. Energy. 2007 Mar 1;72(69):0-399.
102. Harju J, Juvonen P, Kokki H, Remes V, Scheinin T, Eskelinen M. Minilaparotomy cholecystectomy with ultrasonic dissection versus conventional laparoscopic cholecystectomy: a randomized multicenter study. Scandinavian journal of gastroenterology. 2013 Nov 1;48(11):1317-23.
103. Harju J, Aspinen S, Juvonen P, Kokki H, Eskelinen M. Ten-year outcome after minilaparotomy versus laparoscopic cholecystectomy: a prospective randomised trial. Surgical endoscopy. 2013 Jul 1;27(7):2512-6.
104. Dirksen CD, Schmitz RF, Hans KM, Nieman FH, Hoogenboom LJ, Go PM. Ambulatory laparoscopic cholecystectomy is as effective as hospitalization and from a social perspective less

expensive: a randomized study. Nederlands tijdschrift voor geneeskunde. 2001 Dec 15;145(50):2434.

105. Hollington P, Toogood GJ, Padbury RT. A prospective randomized trial of day-stay only versus overnight-stay laparoscopic cholecystectomy. Australian and New Zealand Journal of Surgery. 1999 Dec;69(12):841-3.

106. Johansson M, Thune A, Nelvin L, Lundell L. Randomized clinical trial of day-care versus overnight-stay laparoscopic cholecystectomy. British Journal of Surgery. 2006 Jan 1;93(1):40-5.

107. Young J, O'Connell B. Recovery following laparoscopic cholecystectomy in either a 23 hour or an 8 hour facility. Journal of Quality in Clinical Practice. 2001 Mar;21(1-2):2-7.

108. Plaisier PW, Van der Hul RL, Nijs HG, Den Toom R, Terpstra OT, Bruining HA. The course of biliary and gastrointestinal symptoms after treatment of uncomplicated symptomatic gallstones: results of a randomized study comparing extracorporeal shock wave lithotripsy with conventional cholecystectomy. American Journal of Gastroenterology. 1994 May 1;89(5).

109. Vethrus M, Søreide O, Solhaug JH. Symptomatic, non-complicated gallbladder stone disease. Operation or observation. Scand J Gastroenterol. 2002;37:834-9.

110. Vetrhus M, Søreide O, Nesvik I, Søndenaa K. Acute cholecystitis: delayed surgery or observation. A randomized clinical trial. Scandinavian journal of gastroenterology. 2003 Sep;38(9):985-90.

111. Russell, M. L., Preshaw, R. M., Brant, R. F., Bultz, B. D., & Page, S. A. (1996). Disease-specific quality of life: the Gallstone Impact Checklist. *Clinical and Investigative Medicine. Médecine Clinique Et Experimentale*, *19*(6), 453-460.

112. Urbach, D., Harnish, J., McIlroy, J., & Streiner, D. (2006). A measure of quality of life after abdominal surgery. *Quality of Life Research*, *15*(6), 1053-1061.

113. Chen TY, Landmann MG, Potter JC, et al. Questionnaire to aid priority and outcomes assessment in gallstone disease. ANZ J Surg 2006;76:569-74. 23.

114. Eypasch, E., ood-Dauphin e, S., Williams, J. I., Ure, B., Neugebauer, E., & Troidl, H. (1993). The Gastrointestinal Quality of Life Index. A clinical index for measuring patient status in gastroenterologic surgery. *Der Chirurg,64*(4), 264-74.

115. Lindseth GN, Denny DL. Patients' experiences with cholecystitis and a cholecystectomy. Gastroenterol Nurs. 2014 Nov-Dec;37(6):407-14

116. Kleinbeck SV, Hoffart N. Outpatient recovery after laparoscopic cholecystectomy. AORN J. 1994 Sep;60(3):394, 397-8, 401-2.

117. Psaila J, Agrawal S, Fountain U, Whitfield T, Murgatroyd B, Dunsire MF, Gonzalez JG, Patel AG. Day-surgery laparoscopic cholecystectomy: factors influencing same-day discharge. World J Surg. 2008 Jan;32(1):76-81

118. Peron A, Schliemann AL, Almeida FA. Understanding the reasons for the refusal of cholecystectomy in patients with cholelithiasis: how to help them in their decision? Arq Bras Cir Dig. 2014 Apr-Jun;27(2):114-9.

119. McKneally MF, Ignagni E, Martin DK, D'Cruz J. The leap to trust: perspective of cholecystectomy patients on informed decision making and consent. J Am Coll Surg. 2004 Jul;199(1):51-7.

120. Barthelsson C, Lützén K, Anderberg B, Nordström G. Patients' experiences of laparoscopic cholecystectomy in day surgery. J Clin Nurs. 2003 Mar;12(2):253-9

121. Keus F, de Vries J, Gooszen HG, van Laarhoven CJ. Assessing factors influencing return back to work after cholecystectomy: a qualitative research. BMC Gastroenterol. 2010 Jan 27;10:12

122. Ros E, Zambon D. Postcholecystectomy symptoms. A prospective study of gall stone patients before and two years after surgery. Gut. 1987 Nov;28(11):1500-4.

123. Alkhaffaf B, Blazeby JM, Williamson PR, Bruce IA, Glenny AM. Reporting of outcomes in gastric cancer surgery trials: a systematic review. BMJ Open. 2018 Oct 17;8(10):e021796.

124. Young AE, Davies A, Bland S, Brookes S, Blazeby JM. Systematic review of clinical outcome reporting in randomised controlled trials of burn care. BMJ Open. 2019 Feb 15;9(2):e025135.

For peer review only - http://bmjopen.bmj.com/site/about/guidelines.xhtml

125. Klokker, L., Berthelsen, DB.; Woodworth, T. et al.Identifying Possible Outcome Domains from Existing Outcome Measures to Inform an OMERACT Core Domain Set for Safety in Rheumatology Trials By: Conference: Outcome Measures in Rheumatology Conference (OMERACT) Location: Terrigal, AUSTRALIA Date: MAY 15-18, 2018

126. Duncan, R.; Essat, M.; Jones, G.; et al. Systematic review and qualitative evidence synthesis of patient-reported outcome measures for abdominal aortic aneurysm. B :

127. Shilling, V., Matthews, L., Jenkins, V. Patient-reported outcome measures for cancer caregivers: a systematic review. Quality of Life Resarrch. 2016. 25(8):1859-1876.

128. Harman NL, Wilding JPH, Curry D on behalf of the SCORE-IT Study Team*, et al.* Selecting Core Outcomes for Randomised Effectiveness trials In Type 2 diabetes (SCORE-IT): a patient and healthcare professional consensus on a core outcome set for type 2 diabetes. *BMJ Open Diabetes Research and Care* 2019;7:e000700.

For peer review only

For peer review only - http://bmjopen.bmj.com/site/about/guidelines.xhtml

Authors contributions

	MC	RN	JB	IA	MB	MBr	BC	KI	CR	KG
Conceptualisation									■	
Methodology	■	■	■	■		■			■	■
Investigation	■	■	■		■		■			■
Formal Analysis	■	■	■	■	■				■	■
Writing – Original draft	■	■								■
Writing – Review and Editing	■	■	■	■	■			■	■	■
Visualisation	■	■								■
Supervision										■
Project Administration	■	■						■		■
Funding Acquisition			■	■		■	■		■	■

Funding statement

This work was supported by the National Institute for Health Research (NIHR) Health Technology Assessment (HTA) Programme grant [14/192/71]. The work was also supported by an NHS Grampian Endowment grant [16/11/006]. KG held a Medical Research Council UK Methodology Fellowship during the delivery of this project [MR/L01193X/1]. The Health Services Research Unit, Institute of Applied Health Sciences (University of Aberdeen), is core-funded by the Chief Scientist Office of the Scottish Government Health and Social Care Directorates. The funders had no involvement in study design, collection, analysis and interpretation of data, reporting or the decision to publish.

Competing interests

JB is a member of the COMET Initiative Management Group. No others to declare.

Provenance and peer review

Not commissioned; externally peer reviewed.

Data sharing statement

No further data are available.

For peer review only

Figure 1: PRISMA systematic review diagram for evidence synthesis. PRISMA= Preferred Reporting Items for Systematic Reviews and Meta-Analyses

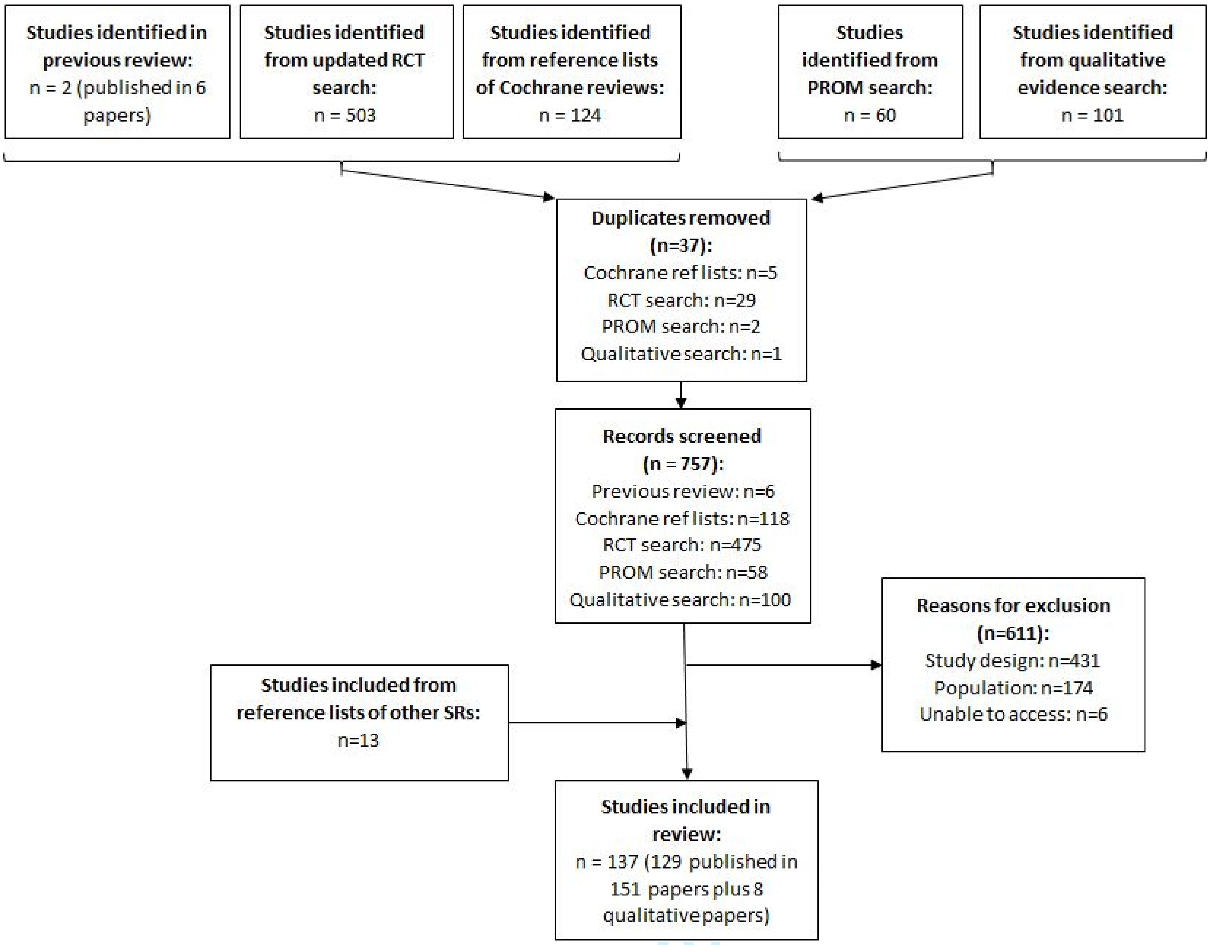

For peer review only - http://bmjopen.bmj.com/site/about/guidelines.xhtml

Table 1: Summary characteristics and demographics of included studies

Characteristic	Quantitative review	Qualitative review
Number of study participants	(n=119 studies)	n=8 studies
Median	75	19.5
Range	14-618	6-100
Total	10757	256
No of males/females	(n=96 studies)	(n=7 studies)
Median	21/53.5	4/16
Range	0-255/15-415	2-15/4-37
Total	2632/6166	43/113
Mean age (years)	(n=14 studies)	-
Median (years)	46.1	-
Range (years)	40-53	19-81
Country	(n=128 studies)	(n=8 studies)
UK	7[a]	1
EEA (excl UK)	44[a]	3
USA	12[a]	1
Other	66	3
No of Centres	(n=129 studies)	(n=8 studies)
Single centre	113	8
Multicentre	16	
Total no of outcomes reported	330	17

[a]One study (Marks 2013) was conducted in the UK, USA and Italy and is included in the count for each country; Number of studies (n=xx studies) in table relates to how many studies reported each relevant characteristic

For peer review only

Table 2 Outcomes reported in randomised controlled trials of interventions to treat uncomplicated gallstone disease

Domain	Outcome	Number of times reported
**Physical**	Physical activity postop	1
	Recovery of self-reported physical activity	1
	Time to resume normal physical activity	3
	Time to resume walking	3
	Functional impairment	1
**Role**	Time to everyday life	1
	Return to daily activities	1
	Return to normal function	1
	Return to activities of daily living	1
	Days from surgery to normal activity	3
	Time to return to normal activities	1
	Days to full activity	1
	No of days sick leave	3
	Time away from work	2
	Loss of active days of work	1
	Time to return to work after discharge	1
	Time to return to work	8
	Days to return to school	1
	Convalescence time	1
	Postop regain of functionality	1
	Total recuperation period	1
	Mobilisation	1
**Pain**	Overall pain	2
	Pain	2
	Visual analogue pain score	2
	Site of most severe pain	1
	Umbilical pain	1
	Abdominal pain	9
	Right upper quadrant pain attacks	3
	Shoulder pain	4
	Admission due to pain	2
	Incisional pain	10
	Whether had pain or other symptoms in incision area/port sites	1
	Patient-reported location of pain at initial postop visit	1
	Postoperative pain	72
	Chronic post surgery pain	1
	Analgesic requirements	58
	Duration of analgesia use	2
	Time to first analgesics	1
	Days of medication required	1
	Analgesia requirement	1
	No of patients needing additional analgesia	1

For peer review only - http://bmjopen.bmj.com/site/about/guidelines.xhtml

	Use of peripheral analgesics	1
	Use of centrally acting analgesia	1
	Total pethidine (mg)	1
	Pethidine requirement	1
	Suppository requests	1
**Bowel movements**	Return of bowel function	1
	Change in bowel habit	1
	Diarrhoea or loose stools	2
	Time to resume passing stools	1
**Thirst/dehydration**	Resumption of oral diet within 24h	1
	Resumption of orals	1
	Resumption of oral intake	3
	Normal drinking	1
	Return to liquid feeds	1
	Time to clear liquids	1
**Appetite/eating/taste**	Time to resume eating	4
	Normal eating	1
	Return to solid diet	2
	Time to resume feeding	1
**Fatigue**	Fatigue	2
**Sleep**	Length of night sleep	1
	Awakening during the night	1
**Generic health**	Overall health state	1
	Health status	1
	Satisfaction with life in general	1
	Patient satisfaction	2
	Overall satisfaction	2
	Satisfaction score	1
	Global patient satisfaction	1
	Patient overall satisfaction	1
	Time interval between onset of symptoms and admission to hospital	1
**Dietary habits**	Need to avoid fried/fatty foods after surgery	3
**Social**	Time away from recreational activity	1
	Return to going out (days)	1
**Belching/Bloating/Gas**	Time to resume passing intestinal gases	1
	Flatulence and/or dyspepsia	1
**Vomiting/nausea**	Nausea or vomiting	5
	Vomiting	7
	Nausea	8
**Reflux**	Reflux symptoms	2
	Heartburn or regurgitation	1
**Regurgitation**	Heartburn or regurgitation	1
**Body Image**	Satisfaction with body image	3
	Patient derived body image	1
	Satisfaction with aesthetic result	1
	Score on body image scale	2
	Wound satisfaction	2
	Satisfaction with cosmetic outcome	10
	Incision satisfaction	1

For peer review only

	Patient satisfaction score on surgery and scars	2
	Cosmetic satisfaction of surgical scar	2
	Patient satisfaction score on scars	1
	Cosmetic outcome	13
	Score on cosmetic scale	1
	Cosmesis	5
	Cosmetic score	1
	Cosmetic result	6
	Incisional cosmesis	1
	Incision cosmetic result	1
	Scar evaluation	4
	Appearance of each incision	1
**Sexual function**	Satisfaction in the context of sexual intercourse	1
	Pain in the context of sexual intercourse	1
	Dyspareunia	1
**Generic symptoms**	Quality of life	22
	Whether operation had had any impact on QoL	1
	Morbidity	8
	Overall morbidity following diagnosis	1
	General discomfort	1
	Symptoms	1
	Symptoms during waiting period	2
	Residual abdominal symptoms	3
	Examined for residual symptoms	1
	Gastrointestinal complaints	1
	Failure of conservative treatment	4
	Failure of technique	1
	No of patients recovered	1
	Complication rates during waiting time for elective laparoscopic cholecystectomy	1
	Success rate	2
**Mortality**	Mortality	30
**Intra-operative adverse events**	CBD stones	1
	CBD injury	3
	Biliary leak	1
	Bile leakage	2
	Biliary injury	1
	Bile duct injury	2
	Bile spillage	1
	Bile duct lesions	1
	Haemorrhage	1
	Blood loss	30
	Rate of intraoperative bleeding	1
	Intraoperative bleeding	1
**Intra and post-operative adverse events**	Adverse events	6
	Intra- and postoperative complications	1
	Operative complications	43
	Complications	6
	Other complications	1
	General complications	2

For peer review only - http://bmjopen.bmj.com/site/about/guidelines.xhtml

	Global complications	1
	Major complications	1
	Abdominal wall complications	1
	Port-site complications	1
	Tissue damage	1
	Intra-abdominal collections	1
**Post operative adverse events**	Postoperative complications	66
	Parietal complications after surgery	2
	Postoperative infection rate	1
	Wound infection(s)	7
	Rate of wound infection	1
	Surgical site infection	1
	Incision infection	1
	Postop wound-related infection	1
	Wound condition	1
	Postop wound-related hernia	1
	Port hernia	2
	Postoperative hernia	1
	Incisional hernia occurrence	6
	Trocar herniation	2
	Intra-abdominal adhesions	1
	Satisfaction with surgery in general	1
	Patient satisfaction score on surgery	2
	Satisfaction with operation	5
	Satisfaction with overall procedural result	1
	Patient satisfaction after surgery	1
	Perceived success of operation	1
	Recommends the procedure	3
	Patient preference survey including "willingness to pay for SILC" questionnaire	1
	Procedure believed to have undergone	1
**Service use**	Readmission before elective operation	1
	Operating time	103
	Operative data	2
	Surgical time	2
	Extensions	1
	Procedure time	1
	System setup time	1
	Performance time	1
	Duration of each operative stage	1
	Learning curve for operating time	1
	Duration of admission	1
	Hospital stay	60
	Postoperative hospital stay	23
	Day surgery	2
	Successful completion of day surgery	3
	Reasons for overnight stay in patients scheduled for day surgery	1
	PACU length of stay	1
	Discharge > 24h postop	1

For peer review only - http://bmjopen.bmj.com/site/about/guidelines.xhtml

	Discharge from hospital	1
	Discharge from hospital 20-24h postop	1
	Time until discharge	1
	Time from operation to discharge	1
	Grade of surgeon	1
	Grade of operating surgeon	1
	Resident's participation	1
	Need to contact hospital or other health care providers after discharge	2
	Ambulatory rate	1
	No of patients requiring readmission to hospital	5
	Readmission	3
	Causes of hospitalisation	2
	No of patients requiring re-intervention	2
	Additional procedures	1
	Reoperation	1
	Reintervention required	1
	Revision surgery	1
	Conversion to open surgery	71
	Conversion of 5mm to 10mm port	2
	Modification of operative technique	4
	Conversion to LC	16
	Conversion to LESS procedure	1
	Conversion to other laparoscopic approach	4
	Conversion	2
	Conversion from SILC to 4PLC	2
	Rate of cholecystectomy	2
**Cost-effectiveness**	Cost of operation	6
	Cost analysis	1
	Procedural cost	1
	Total encounter cost	1
	Hospital cost	5
	Hospital charges	1
	Total cost	1
	Costs	1
	Charge data	1
	Cost-effectiveness ratio	1
	Economic analysis of early versus conventional management for newly diagnosed GB disease	1
**Process (operation or trial)**	Pulmonary function	5
	Spirometric indices	1
	Total Co2 insufflation (litres)	1
	Surgical stress response	2
	No of cannulas used	1
	Pressure of the pneumo peritoneum	1
	Heart rate variability	1
	Serum cytokines	1
	Serum interleukin-6	3
	Serum C-reactive protein	3
	Blood count	1

For peer review only - http://bmjopen.bmj.com/site/about/guidelines.xhtml

	Liver function tests	1
	Alpha-defensins expression	1
	hsCRP values	1
	CRP values	1
	Electroconductivity of representative dermatones	1
	Central & peripheral temperature	1
	Blood examination	1
	Histological findings	1
	Histopathologic findings	1
	Histopathological diagnosis	1
	Occlusive bandages in place	1
	General anaesthesia	1
	Anaesthetic time	1
	Amount of bupivacaine used	1
	Length of skin incision	9
	Extra skin incisions required	1
	Aponeurosis wound size	1
	Wound length	3
	Scar length	2
	Intraoperative cholangiography performed	3
	Intraoperative technical performance	1
	Intraoperative diagnosis	1
	Intraoperative findings	3
	Postop metabolic & hormonal levels	1
	Postoperative level of 8-epiPGF2α	1
	Postoperative level of uric acid	1
	Postoperative forced expiratory volume in 1 second	1
	Change of antibiotic therapy due to nonresponse or to intolerance of moxifloxacin	1
	Use of rescue therapy with 125mg lysine clonixinate tablets	1
	Time of surgical dissection	1
	Time of closure	1
	Detailed surgical time course	1
	Rate of operative error	1
	Technical problems	2
	No of admissions between 8am & 5pm	1
	Admission on Monday to Thursday	1
	Difficulty of case	2
	Operative difficulty	1
	Surgical difficulty	1
	Difficulty of dissection	1
	Difficulty (impaired) of exposure	1
	Prediction of LC difficulty	1
	Surgeon's perceptions of difficulties during operation	1
	Potential for increased surgical risk to the patient	1
	Critical view of safety	1
	Assessment of surgical handling	1
	Feasibility and safety of SILC vs 4PLC	1

For peer review only

For peer review only - http://bmjopen.bmj.com/site/about/guidelines.xhtml

	Ultrasonography findings	1
	No of stones	1
	Size of largest stone	1
	Rate of GB rupture	1
	GB wall thickness	1
	Time to retrieve GB	1
	Number & type of instruments	1
	Use of extra umbilical rescue device 2.3mm mini-LC instrument	1
	Insertion points	1
	Number of incisions	1
	Port sizes	1
	Additional ports required	9
	Port enlargement for GB removal	1
	Additional trocars used	2
	Fourth trocar added	2
	Trocar use	1
	Placement of any additional laparoscopic ports other than the SILS port	1
	Time required for insertion of SILS port system compared to 4 standard ports	2
	Success rate (3-port vs 4-port LC)	2
	Need to amplify extraction port	2
	No of ports used	1
	Reason for port placement	1
	Time of trocar introduction	1
	Use of Keith needle	1
	Successful completion of needlescopic operation	1
	No of drains placed	1
	Requirement of drains	1
	No of manual camera corrections	1
	No of cleaning of the optics	1
	Ability to achieve optimal focus on the operative site during the procedure	1
	Positioning accuracy of image	1
	No of clearing camera	1
	Time of adjusting operative field	1
	Surgeon's comfort	1
	Comfort of instrument positioners	1
	No of actions in positioning of laparoscope	1
	Feasibility of surgical procedures using only the camera robot	1
	Commands misunderstood	1
	No of commands issued by the surgeon	1
	Sum of operative actions	1
	No of dissection actions	1
	No of grasping actions	1
	Time from admission to ultrasonography	1
	Time from ultrasonography to recruitment	1
	Time from recruitment to operation	1

For peer review only

For peer review only - http://bmjopen.bmj.com/site/about/guidelines.xhtml

	Time from recruitment to discharge	1
	Completion of randomised treatment	2
	Gallstone-associated events after randomisation	2

For peer review only

For peer review only - http://bmjopen.bmj.com/site/about/guidelines.xhtml

Table 3. Inclusion of domains across included PROMs ranked by frequency of domains.

Domain (n=22)	No. items per domain	ASIS[112]	CSQ[113]	GIC[111]	GIQLI[114]	*TOTAL*
**Emotional**	17	✓	✓	✓	✓	4
**Pain**	17	✓	✓	✓	✓	4
**Role**	11	✓	✓			2
**Bowel movements**	8	✓			✓	2
**Belching/Bloating/Gas**	7			✓	✓	2
**Appetite/Eating/Taste**	6	✓		✓	✓	3
**Dietary habits**	6		✓	✓		2
**Physical**	6	✓			✓	2
**Fatigue**	5	✓	✓	✓	✓	4
**Social**	4		✓	✓	✓	3
**Sleep**	3	✓			✓	2
**Stomach problems**	3		✓	✓		2
**Generic health**	2		✓		✓	2
**Reflux**	2			✓		1
**Vomiting/nausea**	2			✓		1
**Body Image**	1				✓	1
**Cognitive**	1	✓				1
**Dysphagia/swallowing**	1				✓	1
**Regurgitation**	1				✓	1
**Service use**	1			✓		1
**Sexual function**	1				✓	1
**Thirst/dehydration**	1	✓				1
TOTAL	106	*10*	*8*	*11*	*14*	

For peer review only

For peer review only - http://bmjopen.bmj.com/site/about/guidelines.xhtml

Table 4. Outcomes reported in PROMs, qualitative evidence synthesis and primary qualitative research

Outcome	PROMs	Evidence synthesis	Primary research
Emotional	✓	✓	✓
Pain	✓	✓	✓
Role	✓	✓	
Bowel movements	✓	✓	✓
Belching/Bloating/Gas	✓	✓	✓
Appetite/Eating/Taste	✓		
Dietary habits	✓	✓	✓
Physical	✓		
Fatigue	✓	✓	✓
Social	✓	✓	✓
Sleep	✓	✓	✓
Stomach problems	✓	✓	✓
Generic health	✓		
Reflux	✓	✓	✓
Vomiting/nausea	✓	✓	✓
Body Image	✓		
Cognitive	✓		
Dysphagia/swallowing	✓		
Regurgitation	✓		
Service use	✓		✓
Sexual function	✓		
Thirst/dehydration	✓		
Dizziness		✓	
Fainting		✓	
Prevention of additional disease		✓	
Trust		✓	
Weight		✓	
Cough			✓
Mortality			✓
Problems with breathing			✓
**TOTAL**	22	17	17
**TOTAL INDIVIDUAL OUTCOMES**		**30**	

For peer review only

Table 5. Outcomes Map for uncomplicated gallstone disease

Outcome Domains	Outcome Types	Definitions	Contributing evidence source
Physical	Physical activity	Activities such as walking, running, swimming, cycling, physical labour, climbing stairs, gardening etc	1, 2
	Exercise	Being able to do activities requiring physical effort, carried out to sustain or improve health and fitness (strength and endurance)	3
Role	No. of days sick leave	Length of time off work after the operation in days	1, 2, 3
	Time to everyday life	Length of time taken to return to usual everyday activities	1, 2, 3, 4
	Impact on others	Impact of your gallstone condition or your gallstone surgery on relationships with people surrounding you	2, 3, 4
Pain	Overall pain	Overall pain	1, 2, 3, 4
	Abdominal pain	General pain occurring at rest and/or when coughing, originating in the abdominal area	1, 2, 3, 4
	Umbilical pain	Pain around the belly button scar (this is where the main port is that removed the stones)	1
	Shoulder pain	Pain relating to or affecting the right shoulder region	1, 2
Bowel movements	Diarrhoea	Watery stools, loose bowel motion	1, 2, 3, 4
	Constipation	Difficulty passing stool	2, 3, 4
Thirst/dehydration	Resumption of orals	Starting to eat and drink after treatment	1, 2
Appetite/eating/taste	Time to resume eating	Length of time taken to return to oral food intake	1, 2, 3, 4
Fatigue	Fatigue	Feeling physically or mentally tired or lacking in energy	1, 2, 3, 4
Sleep	Length of night sleep	Length of night's sleep	1, 2, 3, 4
Cognitive	Difficulty concentrating	Inability to focus attention on one task or problem	2
Emotional	Anxiety	A feeling of worry, nervousness or unease	2, 3, 4
	Distress	A feeling of extreme anxiety, stress or anguish	2, 3, 4
	Trust	belief in the reliability, truth, or ability of someone or something	3
Generic health	Quality of life	How well you feel physically and emotionally because of a combination of:<br>Your gallstones<br>The prospect of treatment<br>The result of treatment (Treatment might include surgery or painkillers)	1, 2, 3, 4

For peer review only - http://bmjopen.bmj.com/site/about/guidelines.xhtml

	Overall health state	Overall state of your physical and mental condition	1
	Overall satisfaction	The degree to which expectations or needs have been fulfilled	1
Dietary habits	Food intolerance	A physical adverse reaction by the body to certain foods	1, 4
Social	Time away from recreational activities	Time spent away from enjoyable activities as a result of your gallstone condition or gallstone surgery	1, 2, 3, 4
Belching/bloating/gas	Flatulence	Belching, farting, bloating or gas	1, 2 , 3, 4
	Bloating	Abdominal swelling as a result of excess fluid or gas	2, 3, 4
	Abdominal discomfort	Pain or discomfort in the stomach area	2, 3, 4
Vomiting/nausea	Vomiting	Being sick	1, 2, 3, 4
	Nausea	Feeling sick	1, 2, 3, 4
Reflux	Heartburn	A form of indigestion that presents as a burning sensation in the chest, caused by acid reflux	1, 2, 3, 4
Body image	Satisfaction with body image	A feeling of satisfaction with your own physical appearance	1, 2
	Satisfaction with cosmetic outcome	The extent to which you are content with the cosmetic results of gallstone surgery	1, 2
Sexual function	Satisfaction in the context of sexual intercourse	The extent to which you are satisfied with experiences of sexual intercourse in relation to your gallstone condition or your gallstone surgery	1, 2
	Pain in the context of sexual intercourse	The extent to which you are experiencing pain during or after sexual intercourse in relation to your gallstone condition or your gallstone surgery	1, 2
Regurgitation	Regurgitation	Bringing swallowed food back up to the mouth	1, 2
Dysphagia/swallowing	Trouble swallowing food	Problems swallowing food	2
Generic symptoms	General discomfort	An unpleasant feeling and/or low-level pain which is hard to define	1, 2, 3, 4
	Residual symptoms	Continuing to have symptoms (such as pain, bloating, etc) after removal of the gallbladder	1, 2, 3, 4
	Dizziness	Feeling light headed or dizzy	3
	Fainting	Fainting (short-term loss of consciousness)	3
Mortality	Mortality	Death from any cause	1,4
Intra-operative adverse events	Common bile duct stones	Stones in the common bile duct	1

For peer review only - http://bmjopen.bmj.com/site/about/guidelines.xhtml

	Common bile duct injury	During surgery the common bile duct is damaged	1
	Biliary leak	Liver produces bile which is stored in the gallbladder (see diagram). If this is damaged the bile can leak and cause complications.	1
	Haemorrhage	Bleeding or the abnormal flow of blood; the release of blood from a ruptured blood vessel	1
Intra and post-operative adverse events	Intra-abdominal collections	After surgery any type of fluid collecting in the abdomen.	1
Post operative adverse events	Hernia occurrence	internal hernia – displacement of an organ within the abdomen through a potential defect.	1
	Port-site complications	Complications such as infection, hernia, pain or bleeding at or within the 'keyholes' characteristic of keyhole surgery	1
	Wound infections	An infection at the wound site	1
	Patient perceived success of operation	How patient perceive the success of the operation	1, 3
Service use	Hospital stay	Length of time spent in the hospital from admission to discharge	1, 2,4
Cost-effectiveness	Hospital cost	Total hospital costs, taking into account the total length of hospital stay, operating room charges, medical and surgical supplies, pharmacy, laboratory and pathology, recovery room, anaesthesia, and ICU/observation rooms	1
	Overall cost	Cost of use of healthcare services; e.g. contact with a GP, in- or out-patient contact, prescribed medications	1
	Cost-effectiveness ratio	Cost-effectiveness of treatment route (medical management or surgery to remove the gallbladder), calculated by dividing cost by success rate (defined by the quality of life after treatment)	1

1. Trials of interventions for gallstone disease
2. Patient reported outcome measures
3. Qualitative evidence synthesis
4. Primary qualitative research

For peer review only

APPENDIX 1

UPDATED SEARCH STRATEGY TO IDENTIFY CLINICAL EFFECTIVENESS STUDIES

Ovid MEDLINE(R) Epub Ahead of Print, In-Process & Other Non-Indexed Citations, Ovid MEDLINE(R) Daily and Ovid MEDLINE(R) **1946 – 3rd May 2016** Embase **1980 to 2016 Week 18**

Date of Search: 3rd May 2016

1 cholecystitis/

2 cholecystitis, acute/

3 cholecystolithiasis/

4 gallstones/

5 cholelithiasis/

6 biliary colic/

7 (gall?bladder adj3 (empyema or inflam$)).tw.

8 (biliary colic or gall?stone$ or cholecystitis or cholecystolithiasis).tw.

9 ((pain or biliary symptom$) adj5 (cholecystitis or cholecystolithiasis or gall?bladder)).tw.

10 or/1-9

11 exp Cholecystectomy/

12 cholecystectom$.tw.

13 ((excis$ or remov$) adj4 gall?bladder).tw.

14 ((surgery or surgical) adj5 (cholecystitis or cholecystolithiasis or gall?bladder)).tw.

15 or/11-14

16 exp clinical trial/

17 randomized controlled trial.pt.

18 controlled clinical trial.pt

19 randomi?ed.ab.

20 randomly.ab.

21 trial.ab.

22 placebo.ab.

23 drug therapy.fs.

24 groups.ab.

25 comparative study/

26 (prospective$ or retrospective$).tw.

27 (compare$ or compara$).ti,ab.

28 or/16-27

29 10 and 15 and 28

30 (review or editorial or case report$ or letter).pt.

31 29 not 30

32 limit 31 to human

SEARCH STRATEGY TO IDENTIFY PROMS IN CHOLECYSTITIS

Ovid MEDLINE(R) Epub Ahead of Print, In-Process & Other Non-Indexed Citations, Ovid MEDLINE(R) Daily and Ovid MEDLINE(R) **1946 – 3rd May 2016** Embase **1980 to 2016 Week 18**

Date of Search: 3rd May 2016

1. exp cholecystitis/
2. cholecystolithiasis/
3. gallstones/
4. biliary colic/
5. (gall?bladder adj3 (empyema or inflam$)).tw,kw.
6. (biliary colic or gall?stone$ or cholecystitis or cholecystolithiasis).tw,kw.
7. ((pain or biliary symptom$) adj5 (cholecystitis or cholecystolithiasis or gall?bladder)).tw,kw.
8. or/1-7
9. (core adj3 outcome?).tw,kw.
10. (patient reported adj3 outcome?).tw,kw.
11. prom.tw,kw.
12. 8 and (9 or 10 or 11)
13. *outcome assessment/
14. *"Outcome Assessment (Health Care)"/
15. 8 and (13 or 14)
16. 12 or 15

SEARCH STRATEGY TO IDENTIFY QUALITATIVE STUDIES

Epub Ahead of Print, In-Process & Other Non-Indexed Citations, Ovid MEDLINE(R) Daily and Ovid MEDLINE(R) <1946 to 2nd May 2016

Date of Search: 18th of August, 2016

1. exp cholecystitis/
2. cholecystolithiasis/
3. gallstones/
4. exp Cholecystectomy/

For peer review only

5. biliary colic/

6. (gall?bladder adj3 (empyema or inflam$)).tw,kw.

7. (biliary colic or gall?stone$ or cholecystitis or cholecystolithiasis).tw,kw.

8. ((pain or biliary symptom$) adj5 (cholecystitis or cholecystolithiasis or gall?bladder)).tw,kw.

9. or/1-8

10. qualitative research/

11. exp interviews as topic/

12. focus groups/

13. grounded theory/

14. (qualitative or interview$ or focus group?).tw,kw.

15. (ethno$ or grounded or thematic or interpretive or narrative or discourse analysis or discursive or mixed method$).tw,kw.

16. or/10-15

17. 9 and 16

18. exp animals/ not human/

19. 17 not 18

For peer review only

Appendix 2 . Demographics of participants from primary research

**Research type and number of participants** (n)	**Gender**	**Approached to take part in the CGALL trial**<br>Yes/No and number (n)	**Trial consenters (allocated to receive surgery or medical management) or decliners**
Interviews<br>(n=6)	Female=6	Yes (n=6)	Allocated surgery=4<br>Allocated medical management=1<br>Trial non-consenter=1
Audio-consultations<br>(n=20)	Female= 16<br>Male=4	Yes (n=20)	Allocated surgery= 4<br>Allocated medical Management=3<br>Trial non-consenter=13
Focus Groups<br>(n=5)	Female=4<br>Male=1	No	Not Applicable

For peer review only

BMJ Open

Identification and categorisation of relevant outcomes for symptomatic uncomplicated gallstone disease: in depth analysis to inform the development of a core outcome set

Journal:	*BMJ Open*
Manuscript ID	bmjopen-2020-045568.R1
Article Type:	Original research
Date Submitted by the Author:	20-Feb-2021
Complete List of Authors:	Cruickshank, Moira; University of Aberdeen Institute of Applied Health Sciences, Health Services Research Unit<br>Newlands, Rumana; University of Aberdeen Institute of Applied Health Sciences, Health Services Research Unit<br>Blazeby, Jane; University of Bristol Department of Social Medicine, Department of Social Medicine<br>Ahmed, Irfan; NHS Grampian,<br>Bekheit, Mohamed; NHS Grampian; Department of Surgery, ElKabbary Hospital<br>Brazzelli, Miriam; University of Aberdeen Institute of Applied Health Sciences, Health Services Research Unit<br>Croal, Bernard; Grampian University Hospitals NHS Trust, Clinical Biochemistry<br>Innes, Karen; University of Aberdeen Institute of Applied Health Sciences, Health Services Research Unit<br>Ramsay, Craig; University of Aberdeen, Health Services Research Unit<br>Gillies, Katie; University of Aberdeen Institute of Applied Health Sciences, Health Services Research Unit
**Primary Subject Heading**:	Surgery
Secondary Subject Heading:	Gastroenterology and hepatology, Medical management
Keywords:	Adult gastroenterology < GASTROENTEROLOGY, Gastroenterology < INTERNAL MEDICINE, SURGERY

SCHOLARONE™
Manuscripts

I, the Submitting Author has the right to grant and does grant on behalf of all authors of the Work (as defined in the below author licence), an exclusive licence and/or a non-exclusive licence for contributions from authors who are: i) UK Crown employees; ii) where BMJ has agreed a CC-BY licence shall apply, and/or iii) in accordance with the terms applicable for US Federal Government officers or employees acting as part of their official duties; on a worldwide, perpetual, irrevocable, royalty-free basis to BMJ Publishing Group Ltd ("BMJ") its licensees and where the relevant Journal is co-owned by BMJ to the co-owners of the Journal, to publish the Work in this journal and any other BMJ products and to exploit all rights, as set out in our licence.

The Submitting Author accepts and understands that any supply made under these terms is made by BMJ to the Submitting Author unless you are acting as an employee on behalf of your employer or a postgraduate student of an affiliated institution which is paying any applicable article publishing charge ("APC") for Open Access articles. Where the Submitting Author wishes to make the Work available on an Open Access basis (and intends to pay the relevant APC), the terms of reuse of such Open Access shall be governed by a Creative Commons licence – details of these licences and which Creative Commons licence will apply to this Work are set out in our licence referred to above.

Other than as permitted in any relevant BMJ Author's Self Archiving Policies, I confirm this Work has not been accepted for publication elsewhere, is not being considered for publication elsewhere and does not duplicate material already published. I confirm all authors consent to publication of this Work and authorise the granting of this licence.

For peer review only

Identification and categorisation of relevant outcomes for symptomatic uncomplicated gallstone disease: in depth analysis to inform the development of a core outcome set

Cruickshank M [1,†], Newlands R [1,†], Blazeby JM [2], Ahmed I [1, 3], Bekheit M [3,4], Brazzelli M [1], Croal B [1], Innes K [1], Ramsay C [1], Gillies K [1,*]

[†]Equal contributions

[*]Corresponding author: k.gillies@abdn.ac.uk

1. Health Services Research Unit, Health Sciences Building, Foresterhill, Aberdeen, UK
2. Centre for Surgical Research, University of Bristol, Bristol, UK
3. Department of Surgery, Aberdeen Royal Infirmary, NHS Grampian, Aberdeen, UK.
4. Department of Surgery, ElKabbary Hospital, Alexandria, Egypt

For peer review only

Abstract (298 words)

Background: Many completed trials of interventions for uncomplicated gallstone disease are not as helpful as they could be due to lack of standardization across studies, outcome definition, collection and reporting. This heterogeneity of outcomes across studies hampers useful synthesis of primary studies and ultimately negatively impacts on decision making by all stakeholders. Core outcome sets offer a potential solution to this problem of heterogeneity and concerns over whether the 'right' outcomes are being measured. One of the first steps in core outcome set generation is to identify the range of outcomes reported (in the literature or by patients directly) that are considered important.

Objectives: To develop a systematic map that examines the variation in outcome reporting of interventions for uncomplicated symptomatic gallstone disease, and to identify other outcomes of importance to patients with gallstones not previously measured or reported in interventional studies.

Results: The literature search identified 794 potentially relevant titles and abstracts of which 137 were deemed eligible for inclusion. A total of 129 RCTs, four gallstone disease specific PROMs, and eight qualitative studies were included. This was supplemented with data from six individual interviews, one focus group (n=5 participants) and analysis of 20 consultations. A total of 386 individual recorded outcomes were identified across the combined evidence: 330 outcomes (which were reported 1147 times) from trials evaluating interventions, 22 outcomes from PROMs, 17 outcomes from existing qualitative studies, and 17 outcomes from primary qualitative research. Areas of overlap between the evidence sources existed but also the primary research contributed new, unreported in this context, outcomes.

Conclusions: This study took a rigorous approach to catalogue and map the outcomes of importance in gallstone disease to enhance the development of the COS 'long' list. A COS for uncomplicated gallstone disease that considers the views of all relevant stakeholders is needed.

Strengths and limitations

- This outcome map review is the first to describe the heterogeneity in outcome reporting within uncomplicated symptomatic gallstone disease.
- There is a detailed analysis of all reported outcomes from a range of study designs (both primary and secondary) reporting outcomes of clinical and/or patient relevance.
- A mixed-methods approach was used in both collection and analysis of data.
- Only studies reported in English-language were included in the analysis
- Quality assessment of included studies was not conducted as the main purpose of the review was to extract clinical outcomes not to assess intervention effectiveness.

For peer review only

Background

Gallstone disease (cholelithiasis) is one of the most common gastrointestinal disorders worldwide The prevalence of gallstones is approximately 10-15% in adult populations and they are more common in women and people over the age of 40.[1] Approximately 80% of those affected by gallstone disease are asymptomatic and can remain so for many years without requiring treatment. However, around 20% of patients with gallstones become symptomatic and develop gallstone related complications. These patients are offered symptom control and/or surgical/endoscopic intervention.[2] A significant number of these patients remain symptomatic only (i.e. experiencing pain) without developing gallstone related complications.[2]

Recommendations from the recent NICE guideline on Gallstone Disease have clearly demonstrated insufficient information for patients with gallstones on the effect of cholecystectomy on patient outcomes.[3] The Guideline recommends "research is needed to establish the long-term patient benefits and harms, so that appropriate information can be provided to patients to aid decision-making and long-term management of their condition."[3] However, many completed trials are not as helpful as they could be due to lack of standardization across studies, outcome definition, collection and reporting. This heterogeneity of outcomes across studies also hampers useful synthesis of primary studies in meta-analyses and ultimately negatively impacts on decision making by all stakeholders. In addition to the heterogeneity of outcomes currently reported and the problems this causes, measuring the wrong outcomes (i.e. those that are not valued by clinicians or, more importantly, patients) could also be a real risk for many studies if stakeholders are not consulted during the trial design process. One way that these problems with heterogeneity and relevance to stakeholders can be addressed is through the development and use of core outcome sets.[4,5] There is currently no agreed published core outcome set for evaluating interventions to treat symptomatic uncomplicated gallstone disease.

Core outcome sets aim to define a minimum set of outcomes that should be considered essential for the evaluation and reporting of specific interventions or conditions (i.e. the set of outcomes that should always be considered and ideally measured in any evaluation).[4,5] There is a growing body of literature to provide support for development of core outcome sets.[5] Specifically, they are developed using consensus methods involving stakeholder groups, such as health professionals and patients, so as to ensure that the outcomes being defined are both clinically and personally relevant for the individuals involved.[4,5] Assessment of a core outcome set is not expected to be mutually exclusive to the measurement of other outcomes. However, a core set will foster greater consistency in outcome reporting between studies and lead to more meaningful data being available to contribute to meta-analysis.[4,5] Moreover, core outcome sets can minimise the threat of outcome reporting bias by ensuring consistency between what is measured and what is reported.[4,5] Ultimately, they should improve the overall efficiency and quality of the evidence on which health care decisions can be made.

A core outcome set for uncomplicated gallstone disease is currently being developed. Details of this project have been registered and included in the Core Outcome Measures in Effectiveness Trials (COMET) Initiative database.[6] Outcome mapping is an important step in the development of core outcome sets, to present and

For peer review only

catalogue the outcomes reported to date, and links the literature review and the subsequent process of consultation and consensus.[7] Therefore, the objectives of this paper are to document the outcome mapping process in the development of the core outcome set for symptomatic uncomplicated gallstone disease.

Methods

The protocol for development of the COS is available on the COMET website: http://www.comet-initiative.org/studies/details/927?result=true.

Identification of outcomes relevant for symptomatic uncomplicated gallstone disease.

The identification of outcomes was informed by two sources: existing evidence and new primary research. The specifics of these are detailed below.

1. Identification of outcomes from existing literature:
    a. Outcomes reported in trials of interventions for symptomatic uncomplicated gallstone disease.
    b. Content analysis of individual items within disease specific Patient Reported Outcome Measures (PROMs).
    c. Outcomes from exploratory studies reported by patients with a lived experience of symptomatic uncomplicated gallstone disease.
2. Identification of outcomes of relevance to patients from new primary research.
    a. Interviews and focus groups with patients with a diagnosis of symptomatic uncomplicated gallstone disease and a range of treatments planned or received.
    b. Audio-recordings of consultations for a clinical trial evaluating treatments for symptomatic uncomplicated gallstone disease.

Methods for each of these outcome identification stages are specified below.

Identification of outcomes from existing literature: outcomes reported in trials of interventions for symptomatic uncomplicated gallstone disease and identification of disease specific PROMs.

Reported outcomes of interventions for symptomatic uncomplicated gallstone disease were identified by updating the search strategy for a recent systematic review (Brazzelli et al 2015[8]; this review included two RCTs), by conducting a search for relevant PROMs and by screening the reference lists of relevant Cochrane reviews. In addition, reference lists of systematic reviews identified by the search strategy were checked for relevant RCTs.

Search methods for identification of studies

Extensive electronic searches were undertaken to identify trials for a project on the clinical effectiveness of cholecystectomy and these are reported in full elsewhere.[8] The databases searched included MEDLINE (1946 to week 37 2012), MEDLINE-in-process (10 September 2012), Embase (1974 to 2012 September 10), Science Citation Index (1970 to 12 September 2012), BIOSIS (1956 to 12 September 2012) and the Cochrane Central Register of Controlled Trials (Issue 9 of 12, 2012). Studies identified from these searches were used to elicit

For peer review only

reported outcomes. The MEDLINE and EMBASE searches were updated in May 2016 (Sep 2012 – May 2016) to identify more recent relevant trials.

In addition, a specific search of MEDLINE and EMBASE was undertaken to identify studies that report PROM outcome data for cholecystitis, with records retrieved by the main search for trials excluded to avoid duplication of the results. This search was undertaken in May 2016 (1980-May 2016). The search strategies for MEDLINE and EMBASE are reported in Appendix 1. Inclusion criteria for eligible studies were as follows; Participants: Adults aged over 18 years with symptomatic uncomplicated gallstone disease. Intervention and comparator: Any intervention (surgical or non-surgical management, i.e. expectant management or dietary advice, or medical therapy) used to manage symptomatic uncomplicated gallstone disease in adults. Outcomes: All reported outcomes well eligible for inclusion. Excluded studies included those focusing on asymptomatic gallstone disease or on acute severe cholecystitis, cholangitis, or pancreatitis were not considered suitable for inclusion. In addition, studies including 'complex' gallstone cases i.e. empyema, ascending cholangitis and gallstone ileus, were excluded. Reports published in non-English languages for which a translation could not be organised were also excluded. In addition, lists of included and excluded studies reported in several relevant Cochrane reviews were checked by one reviewer (MC) for potentially relevant studies .[9-14]

Study selection and data extraction

One reviewer (MC) screened all titles and abstracts identified by the two search strategies and a second reviewer (RN) checked a 10% random sample. All full text papers considered potentially eligible were screened by one reviewer (MC) and checked by a second reviewer (RN). One reviewer (MC) extracted details of all outcomes reported (verbatim) and any reported definition of outcomes provided by the authors (e.g. operating time may have been defined and reported by some studies as 'interval between initial skin incision and sin closure' others 'duration of surgery' etc. The definition reported by study authors was used to when de-duplicating items into a shorter list. Data was recorded in a Microsoft Excel file. A 10% sample was checked by a second reviewer (RN). Other relevant data (i.e., study and participant characteristics) were extracted by one reviewer (MBe) and checked by a second reviewer (MC). At all stages, disagreement between reviewers was resolved by discussion.

Data extraction from PROMs

From the list of outcomes reported in trials of interventions for symptomatic uncomplicated gallstone disease described above, disease specific PROMS were identified and supplemented with the studies identified in the search. Data were extracted by one reviewer (KG) who recorded the name of the PROM(s), the reported PRO scales and individual verbatim items. The individual verbatim items from each PROM were analysed using an inductive content analysis approach and informed by previous PROM coding work .[15] All PROM items were systematically categorised into conceptual health domains according to the aspect which they aim to capture. Health domains were generated inductively from the identified individual items. Domain

For peer review only

mapping was conducted two authors (KG and JB) independently with any conflicts resolved through discussion.

***Identification of outcomes from existing literature*: *outcomes reported as important by patients with a lived experience of uncomplicated gallstone disease*.**

Search methods for identification of studies

A search for relevant qualitative studies was undertaken in August 2016 in the Ovid versions of MEDLINE (from 1966-2016). The search strategies combined search terms for cholecystitis, cholecystectomy, with terms for qualitative research (Appendix 1). Inclusion criteria included (i) studies that have explored (using observations, interviews, focus groups and other qualitative methods) participants' lived experiences of gallstones with specific reference to outcomes of importance. Exclusion criteria included (i) studies that have not used qualitative methods; (ii) any review articles, conference abstracts and those with no full-text articles published or non-English language articles.

Study selection and data extraction

One researcher (RN) screened all abstracts and another (KG) screening a random 10% sample . Full-text articles were obtained for those that were potentially relevant. Two researchers (RN and KG) reviewed all potentially relevant articles to ensure they met the inclusion criteria. To identify additional relevant studies, the reference lists of the included studies were also examined.

Data on study characteristics such as author, publication date, country, focus of investigation, data collection methods, number of participants, and details on sample size were extracted. Additionally, two authors (KG and RN) independently extracted data from two main sources reporting study findings: 1. Direct quotes from participants and; 2. Authors interpretations of participants quotes. This data was recorded verbatim and analysed to identify 'descriptive' thematic codes. Constant comparison method was used to compare findings across studies and an inductive thematic synthesis was undertaken to generate a list of themes and sub-themes (focused on outcomes) from the data to map across the pre- and post-surgery timeline.[16,17] Throughout this process, the description and wording of the themes were continually revised, and notes made as to how themes and/or sub-themes related and how some could be merged. These findings were discussed further with the research team to finalise the themes across the studies and these were considered, where appropriate, as domains relevant for inclusion in the development of the COS.

Identification of outcomes of relevance to patients from new primary research.

In addition to outcomes reported in existing literature, we conducted primary qualitative research to further inform the identification of outcomes of relevance to patients. Three activities were conducted:

1. Interviews with patients with a diagnosis of symptomatic uncomplicated gallstone disease;
2. A focus group with patients who had undergone cholecystectomy;
3. Analysis of audio-consultations from a trial comparing surgical versus medical management of symptomatic uncomplicated gallstone disease .

For peer review only - http://bmjopen.bmj.com/site/about/guidelines.xhtml

Participant identification and invitation

Potential participants for the interviews were identified from the ongoing CGALL trial and were provided with a study participant information leaflet (PIL) either in the clinic or posted to the participant if a decision about CGALL trial entry was made later.[18] The PIL contained a detachable reply-slip to complete and return to the researcher (in a reply paid envelope) if they would like to discuss participating in the interview study. Patients being approached to participate in the CGALL trial were asked (for trial purposes) if they would consent to their consultation being audio-recorded. If consent was obtained, these audio-recordings were then analysed for the identification of outcomes.

Focus group participants were identified through the Scottish Health Research register (SHARE - https://www.registerforshare.org/) and sent an invitation letter asking them to contact the research team if interested in participating. Following initial contact, a researcher phoned the interested participants and ensured they were clear about what the study entailed and arranged a suitable time for the focus group.

Data collection, management and analysis

One author (RN) conducted the interviews over the telephone between [April - August, 2017] . The focus group was conducted by two members of the trial team (KG and the PPI partner BC) on 20th July, 2017. Trial consultations were conducted as standard and four sites across the trial were sampled to inform outcome identification. Informed (written and recorded) consent was obtained from all participants prior to data collection, and confidentiality of the participants was assured. Participants were encouraged to consider what aspects of their disease or treatment impacted them most, both in terms of physical and psychological functioning and what improvements they would wish to see in terms of outcomes. Interviews, focus group and audio-consultations were audio-recorded and transcribed verbatim using a professional transcription service. All transcripts were imported into NVivo (Version 10, 2013: QSR International) and analysed using conventional content analysis (i.e. coding categories are derived directly from the text data and are used to interpret meaning from the content).[19] Various themes and sub-themes were generated by one researcher (RN) based on the contents of the transcripts to identify the outcomes stated by the participants, and these were then further discussed (with KG) to finalise the list of outcomes identified across the primary qualitative data. The analysis was oriented to address the aim of identifying the range of outcomes that might be considered important and the reasons used to justify assessment of them as important.

Categorisation of identified outcomes into outcome domains

The list of potential outcomes generated from the systematic evidence search and primary qualitative research formed the basis of a 'long' list of outcomes used to refine the items into a final 'short' list for inclusion in the Delphi stage of core outcome set development. Outcomes were first grouped and reduced according to original source, i.e. the initial long list from the evidence review was reduced for duplication by two members of the research team (MC and IA). A similar process was conducted to de-duplicate the outcomes identified from the PROM coding, qualitative evidence synthesis and primary qualitative research

For peer review only - http://bmjopen.bmj.com/site/about/guidelines.xhtml

(KG and RN). These outcomes lists were then merged to identify areas of overlap and reduce for further duplication through iterative group discussions (further addressing duplications and relevance of outcomes for effectiveness trials) to produce a final short list OF individual outcomes of relevance in this context.

Individual outcome items were further grouped into broader concept level headings to categorise outcome domains. These concept level headings were informed by other outcome categorisation work in the area of COS and supplemented through study management group discussion. The categorization was performed by one member of the team (KG) and refined through iterative discussions.[15,20,21]

Ethical approval

The primary qualitative research in this study was approved as part of the CGALL trial by the North of Scotland Research Ethics Service [16/NS/0053] and NHS Grampian Research and Development.

Patient and Public Involvement

The outcome mapping work reported in this manuscript has involved the input of patients from inception, through design, conduct and reporting. Co-author BC is a patient partner and has been involved in all phases of project design and delivery and specifically helped to facilitate the patient focus groups to identify outcomes of importance to patients.

Results

Studies identified from the search of existing literature

The literature search identified 794 (633 from the search of trials, 60 from the PROM search and 101 from the search of qualitative literature) potentially relevant titles and abstracts for screening. Of these, 137 (129 from trials and PROMs and 8 qualitative studies) were deemed eligible for inclusion (Figure 1). A total of 137 publications from 129 RCTs, four gallstone disease specific PROMs, and eight qualitative studies were therefore included

Sample characteristics of existing literature and new primary research

Existing literature

Seven studies were conducted in the UK. A further 44 were conducted in countries in Europe, other than the UK, 12 in the USA and 66 in a range of other countries. A total of over 10000 participants were included in the studies, predominantly women (sex ratio around 2.5:1) in their mid 40s (40-53, median 46.1). Studies were mainly single-centre (n=113) and ranged in size from small (14 participants) to large (618 participants) trials (median n=75). A vast majority of trials involved one type of surgery versus another type of surgery, with single incision laparoscopic cholecystectomy versus conventional laparoscopic cholecystectomy being the most common trial configuration.[22-71] Other common configurations were early laparoscopic cholecystectomy versus delayed laparoscopic cholecystectomy,[72-87] mini laparoscopic cholecystectomy versus laparoscopic cholecystectomy,[88-97] mini laparotomy versus laparoscopic cholecystectomy,[98-103] and day-case laparoscopic cholecystectomy versus overnight stay laparoscopic cholecystectomy.[104-107] There

For peer review only

were few non-surgical interventions; one study compared shock wave lithotripsy and laparoscopic cholecystectomy,[108] whilst several compared observation with cholecystectomy.[109-110] The four disease specific PROMS for uncomplicated gallstone disease were developed in Canada (n=2), .New Zealand (n=1) and Germany (n=1).[111-114] Two of the PROMS focused on gallstone disease,[111,113] one on gastrointestinal diseases,[114] and the other on quality of life after abdominal surgery.[112] All of the PROMs reported to measure multi-dimensional constructs e.g. quality of life. The PROMs varied in the number of constructs they aimed to assess (ranging from 4-8) and the number of items they asked participants to report on (ranging from 5-41, median = 27).

The eight qualitative studies from seven different countries: USA (n=2),[115,116] UK,[117] Brazil,[118] Canada,[119] Sweden,[120] the Netherland[121] and Spain[122] were identified including 324 participants (ranged from 12-162, median = 19.5). They were predominantly women and their age ranged from 19-81 years. Seven studies used interviews either face-to-face (n= 5) or by telephone (n=2) and one used focus groups for data collection. All of the treatments being explored in the studies were surgery but the types of surgery varied. Five studies investigated patients' experiences after surgery, two investigated experiences of cholecystitis (i.e. inflammation of the gall bladder) and surgery and another investigated experiences of cholelithiasis (i.e. presence of gallstones). [115-122] Table 1 provides a summary of the characteristics of participants included in the quantitative and qualitative literature review.

Primary research

Six individual interviews, one focus group (n=5 participants) and analysis of 20 consultations (5 each from 4 different hospital sites) were conducted providing data from a total sample of 31 patients. A brief description of the participants is provided in Appendix 2. They included 26 women and five men, 26 of whom had been approached to take part in the CGALL trial (12 trial consenters and 14 trial non-consenters), and 5 patients whom had not been approached about the trial but who had all had a cholecystectomy. The CGALL trial is a randomised controlled trial comparing laparoscopic cholecystectomy with conservative management for preventing recurrent symptoms and complications in adults with uncomplicated symptomatic gallstones.[18]

Outcomes identified from existing literature and new primary research

A total of 386 individual recorded outcomes were identified across the combined evidence from existing literature and new primary research: 330 outcomes (which were reported 1147 times) from trials evaluating interventions, 22 outcomes from PROMs, 17 outcomes from existing qualitative studies, and 17 outcomes from primary qualitative research.

Of the 330 individual outcomes reported in trials, 97 (29.4%) were reported as 'primary outcomes' with only 64 (19.4%) being formally defined and 227 (68.8%) were reported by one study only. The three "verbatim" outcomes which were reported most frequently were: "operating time" (n=103), "postoperative pain" (n=72) and "conversion to open surgery" (n=71). In addition, two studies reported the outcome "conversion". Four studies reported further outcomes relating to "operating time", i.e. "anesthetic time" (n=1), "surgical time"

For peer review only - http://bmjopen.bmj.com/site/about/guidelines.xhtml

For peer review only

(n=2), “procedure time” (n=1). Forty studies reported further outcomes relating to pain: “umbilical pain” (n=1), “abdominal pain” (n=9), right upper quadrant pain attacks” (n=3), “chronic post-surgery pain” (n=1), “overall pain” (n=2), “incisional pain” (n=10), “pain in the context of sexual intercourse” (n=1), “visual analogue pain score” (n=2), “pain” (n=2), “shoulder pain” (n=4), “pain or other symptoms in incision area/port sites” (n=1), “patient reported location of pain at initial postop visit” (n=1), “site of most severe pain” (n=1), “admission due to pain” (n=2), . Table 2 provides a list, and frequency, of all outcomes reported in the included trials.

Some 106 individual items were identified from the four gallstone disease specific PROMS covering 22 individual outcomes, with frequency of each outcome varying across the individual PROMs (Table 3). Included PROMs covered between eight and 14 domains. None of the included PROMS reported whether patients were involved in the measures development. Pain and emotional outcomes were the most frequently covered with 17 items each (making up 32% of total items) and reported across all four PROMs. There were seven outcomes identified only once (and four of which were identified in one PROM) across the 106 items, which included thirst/dehydration, cognitive, service use, body image, sexual function, regurgitation, swallowing.

Seventeen individual outcomes were identified from the existing qualitative literature (see Table 4). Twelve overlapped with existing outcomes identified in the literature and the PROMS leaving 5 additional outcomes for consideration in the long list, namely: dizziness, fainting, trust, weight, and prevention of additional disease.

The primary qualitative research identified 17 individual outcomes, with the majority (n=14) overlapping with those reported in the previously reviewed evidence (See Table 4). However, three additional outcomes (breathing problems, cough, and mortality) were identified that weren’t in the previous patient focused evidence (PROMs and qualitative literature), with 2 of these (breathing problems, cough) making unique contributions to the overall outcome list.

The 390 individual reported outcomes across the 4 data sources were reduced into a ‘short’ list of outcomes which could be measured in comparative effectiveness trials (i.e. Phase III pragmatic evections trials) of interventions to treat uncomplicated symptomatic gallstone disease (See Table 5). This resulted in several outcomes being dropped from the long list as deemed not eligible as clinical endpoint outcomes for use in trials of this type (e.g. system and process outcomes such as duration of surgery which might be important in earlier phase trials). Therefore, the final list covered 27 broad outcome domains that contained 41 distinct outcomes. The domains of pain, intra-operative complications, and post-operative complications contained the most outcomes (n=4 each).

Discussion

For peer review only

Currently there is a lack of consistency in the selection, measurement and reporting of outcomes for uncomplicated gallstone disease. This leads to challenges in evidence synthesis and decision-making. A Core Outcome Set would be an important step to improve this situation. This paper which describes an outcome mapping exercise is the first comprehensive step in the development of a core outcome set. It catalogues and reports the outcomes that have been measured in trials of interventions to treatment uncomplicated gallstone disease. It extends this initial phase of outcome identification to include outcomes from PROMs, published qualitative evidence, and empirical qualitative research. Over 1000 verbatim outcomes were identified and reduced through de-duplication to 390. This was further reduced to 41 outcomes spanning 27 domains. The next steps in this work are to reach consensus for the COS for uncomplicated symptomatic gall stone disease.

As with many other outcome mapping exercises, this first stage of this study highlights the significant heterogeneity that exists within clinical trials comparing treatments for gallstone disease. Of the 334 outcomes, which were reported multiple times across the 129 RCTs, almost 70% were reported by only one study – a finding comparable with other outcome mapping studies.[21,123,124] All of the effort into collection and reporting of these outcomes is likely wasted as it is doubtful that they could be combined with others to make more confident assessment of the effectiveness of treatments. This outcome heterogeneity in existing trials is further emphasised when considering the outcome of pain, which is reported in 72 trials as post-operative pain but also reported in a number of other trials using 15 different outcomes. The four disease specific PROMs identified further extend the problem of outcome heterogeneity. Whilst all of these measures report to capture quality of life, there is variability in both the inclusion and emphasis of domains captured across each of the tools. For example, the most frequently covered domains capture emotion and pain and are reported across all four tools. Fatigue is also reported across all four tools but is only captured by 5 items as opposed to the 17 each for emotion and pain. Nine of the 22 domains are only captured by one of the PROMs, with a further eight domains being captured by 2 PROMs. Again this variability in what individual items are measured in these tools and how they contribute to overall 'quality of life' assessments, raises questions about the legitimacy of combining these measures when evaluating intervention effectiveness. In addition, the relevance of these outcomes to patients must be called into question given the lack of reporting of input from patients in the item inception phase of PROM design across these measures. Two reviews published after completion of this work conducted an methodological assessments of both disease specific and generic PROMs for laparoscopic cholecystectomy and both report considerable variation and a lack of patient involvement.[125,126] There are now several reviews of PROMs in other clinical specialties that also provide findings which further highlight the heterogeneity that exists across measures which, on the surface, report to measure similar concepts.[20,127-129]

The different evidence sources contributed to the final outcome short list in a variety of ways with the outcomes reported in previous trials often capturing clinically focussed endpoints and the PROMS and qualitative research providing more patient focused outcomes. When considering what outcomes matter to patients and how this contributes to the outcome mapping in this area, this study used two approaches

For peer review only - http://bmjopen.bmj.com/site/about/guidelines.xhtml

to ensure patient relevant outcomes were included. The qualitative evidence synthesis and primary research identified a total of 34 combined (of which 21 were mutually exclusive) outcomes, that were important to patients in terms of their gallstone disease or their perceptions about treatments. Theses outcomes could be broadly grouped into physical and social functioning with most reports from participants focusing on reduction in pain and a desire to 'return to normal'. When compared to the outcome domains reported in the PROMs, there was considerable overlap between the two sources. However, there were some areas of discordance between the different sources, with the qualitative data adding a further 8 outcomes. In addition the qualitative evidence synthesis and the empirical research identified outcomes not previously measured or reported in comparative effectiveness trials for uncomplicated gallstone disease. This value of including evidence from existing literature exploring patients perspective and/or new primary research to identify patient relevant outcomes is gaining traction amongst COS developed.[5] Where most have included outcomes through identification in interviews, the use of qualitative evidence synthesis is growing, and especially in areas where there has previously been a considerable volume of work to draw on.[130] These studies have shown that this work contributes previously unreported outcomes that are of importance to patients and hence underpins the critical nature of this step in COS development. Whether these outcomes identified in these list development stages end up making it into the COS is currently less well evidenced but will be important to know.

Strengths and limitations

This outcome mapping exercise used a systematic search to identify outcomes reported in both quantitative and qualitative studies in the literature. In addition to this rigorous systematic search we supplemented the pool of outcomes already available with new primary qualitative research (through three methods) to further identify outcomes that matter to patients who are experiencing uncomplicates symptomatic gallstone disease. This complementary approach to identification of outcomes has ensured a broad catalogue of both clinically relevant and patient important outcomes. Limitations of this review are linked to the inclusion of only English language studies, a lack of quality appraisal of included studies, and no assessment of reporting bias. Whilst these decisions were fit for purpose for the COS activity, they may have introduced potential reporting and selection bias within the outcome map. With regard to outcome reporting bias, other COS development papers have explored this and found that in surgical studies of oesophagectomy and colorectal cancer resection papers frequently did not report all the outcomes intended to be measured (50% at least or more did not do that). [131,132] Future COS development studies should consider this approach to assess outcome reporting bias. It would also have been useful to collect the study teams reported rationale for the selection of reported outcomes to determine how that process was determined.

Conclusions

This study took a rigorous approach to catalogue and map the outcomes of importance in gallstone disease to enhance the development of the COS 'long' list. The synthesis of data from the four different evidence sources further underpinned the need for a COS in this space due to the heterogeneity of outcome

For peer review only - http://bmjopen.bmj.com/site/about/guidelines.xhtml

measurement and reporting. However, the extensive use of data sources to contribute to the development of the list of outcomes for further consensus agreement, did highlight ‘new’ outcomes that have not been previously reported for trials evaluating interventions for gallstone disease and many of these ‘new’ outcomes were those reported by patients. This comprehensive approach to the development of the long list, and then ultimately the short list for scoring in a COS, gives confidence that both clinically relevant and patient focused outcomes have been considered and have the potential to be represented in the agreed COS.

Acknowledgements

The authors thank the HSRU Information Scientist for help with the database searches and the CGALL trial team and management group for ongoing advice and contribution to the study. We would also like to thank the patients who contributed their time to for the interviews.

(4865 words)

For peer review only

References

1. Barbara L, Sama C, Labate AMM. A population study on the prevalence of gallstone disease: The Sirmione study. *Hepatology* 1987;7:913-7
2. Festi D, Reggiani MLB, Attili AF, Loria P, Pazzi P, Scaioli E, et al. Natural history of gallstone disease: Expectant management or active treatment? Results from a population-based cohort study. *J Gastroenterol Hepatol* 2010;25:719-24
3. Gallstone disease. NICE Clinical Guideline CG188. *National Insititute for Health and Care Excellence* 2014. http://www.nice.org.uk/guidance/cg188 [accessed April 2016]
4. Williamson PR, Altman DG, Blazeby JM, Clarke M, Devane D, Gargon E, Tugwell P. Developing core outcome sets for clinical trials: issues to consider. Trials. 2012 Aug 6;13:132
5. Davis K, Gorst SL, Harman N, Smith V, Gargon E, Altman DG, Blazeby JM, Clarke M, Tunis S, Williamson PR. Choosing important health outcomes for comparative effectiveness research: An updated systematic review and involvement of low and middle income countries. PLoS One. 2018 Feb 13;13(2):e0190695.
6. http://www.comet-initiative.org/studies/details/927?result=true *Last accessed 24/009/20*
7. Kaufman J, Ryan R, Bosch-Capblanch X, Cartier Y, Cliff J, Glenton C, Lewin S, Rada G, Ames H, Muloliwa AM, Oku A, Oyo-Ita A, Hill S. Outcomes mapping study for childhood vaccination communication: too few concepts were measured in too many ways. J Clin Epidemiol. 2016 Apr;72:33-44.
8. Brazzelli M, Cruickshank M, Kilonzo M, Ahmed I, Stewart F, McNamee P, Elders A, Fraser C, Avenell A, Ramsay C. Systematic review of the clinical and cost effectiveness of cholecystectomy versus observation/conservative management for uncomplicated symptomatic gallstones or cholecystitis. Surg Endosc. 2015 Mar;29(3):637-47. doi: 10.1007/s00464-014-3712-6.
9. Gurusamy, K.S., Samraj, K., Fusai, G. and Davidson, B.R., 2008. Early versus delayed laparoscopic cholecystectomy for biliary colic. *Cochrane Database of Systematic Reviews*, (4).
10. Gurusamy, K.S., Koti, R., Fusai, G. and Davidson, B.R., 2013. Early versus delayed laparoscopic cholecystectomy for uncomplicated biliary colic. *Cochrane Database of Systematic Reviews*, (6).
11. Gurusamy, K.S., Davidson, C., Gluud, C. and Davidson, B.R., 2013. Early versus delayed laparoscopic cholecystectomy for people with acute cholecystitis. *Cochrane Database of Systematic Reviews*, (6).
12. Gurusamy, K.S., Samraj, K., Fusai, G. and Davidson, B.R., 2012. Robot assistant versus human or another robot assistant in patients undergoing laparoscopic cholecystectomy. *Cochrane database of systematic reviews*, (9).
13. Gurusamy, K.S., Vaughan, J., Ramamoorthy, R., Fusai, G. and Davidson, B.R., 2013. Miniports versus standard ports for laparoscopic cholecystectomy. *Cochrane Database of Systematic Reviews*, (8).
14. Gurusamy, K.S., Vaughan, J., Rossi, M. and Davidson, B.R., 2014. Fewer-than-four ports versus four ports for laparoscopic cholecystectomy. *Cochrane Database of Systematic Reviews*, (2).
15. Macefield, R.C., Jacobs, M., Korfage, I.J. *et al.* Developing core outcomes sets: methods for identifying and including patient-reported outcomes (PROs). *Trials* **15,** 49 (2014). https://doi.org/10.1186/1745-6215-15-49
16. Hewitt-Taylor J. Use of constant comparative analysis in qualitative research. Nurs Stand. 2001 Jul 4-10;15(42):39-42.
17. Thomas J, Harden A. Methods for the thematic synthesis of qualitative research in systematic reviews. BMC Med Res Methodol. 2008 Jul 10;8:45
18. https://w3.abdn.ac.uk/hsru/C-GALL/Public/Public/index.cshtml *Last accessed 24/009/20*
19. Hsieh HF, Shannon SE. Three approaches to qualitative content analysis. Qual Health Res. 2005 Nov;15(9):1277-88
20. Hopkins, J C; Howes, N; Chalmers, K; Savovic, J; Whale, K; Coulman, K D; Welbourn, R; Whistance, R N; Andrews, R C; Byrne, J P; Mahon, D; Blazeby, J M. Outcome reporting in bariatric surgery: an in-depth analysis to inform the development of a core outcome set, the BARIACT StudyObesity reviews. , 2015, Vol.16(1), p.88-106
21. Coulman KD, Hopkins J, Brookes ST, Chalmers K, Main B, Owen-Smith A, Andrews RC, Byrne J, Donovan JL, Mazza G, Reeves BC, Rogers CA, Thompson JL, Welbourn R, Wordsworth S, Blazeby JM; BARIACT working group. A Core Outcome Set for the Benefits and Adverse Events of Bariatric and Metabolic Surgery: The BARIACT Project. PLoS Med. 2016 Nov 29;13(11):e1002187.
22. Abd Ellatif ME, Askar WA, Abbas AE, Noaman N, Negm A, El-Morsy G, El Nakeeb A, Magdy A, Amin M. Quality-of-life measures after single-access versus conventional laparoscopic cholecystectomy: a prospective randomized study. Surgical endoscopy. 2013 Jun 1;27(6):1896-906.

23. ClinicalTrials.gov. *Safety and Cost-effectiveness Study of Single Port Laparoscopic Cholecystectomies (SPoCOT)*. Bethesda, MD: National Library of Medicine; 2009 [https://clinicaltrials.gov/ct2/show/NCT00974194]
24. Aprea G, Bottazzi EC, Guida F, Masone S, Persico G. Laparoendoscopic single site (LESS) versus classic video-laparoscopic cholecystectomy: a randomized prospective study. Journal of Surgical Research. 2011 Apr 1;166(2):e109-12.
25. Artis T, Kucuk C, Akay A, Zararsiz G, Sozuer E. Prospective randomized study comparing single incision vs standard laparoscopic cholecystectomy. Surg Endosc 2014;28:S1-S53.
26. UMIN Clinical Trials Registry. *Randomized controlled trial to evaluate the superiority of the single port cholecystectomy on reduction of postoperative pain, compared with conventional laparoscopic cholecystectomy*. Japan: University Hospital Medical Information Network; 2009 [https://upload.umin.ac.jp/cgi-open-bin/ctr_e/ctr_view.cgi?recptno=R000003103]
27. ClinicalTrials.gov. *Single port laparoscopic cholecystectomy versus four port laparoscopic cholecystectomy: impact on postoperative pain*. Bethesda, MD: National Library of Medicine; 2011 [https://clinicaltrials.gov/ct2/show/NCT01348620]
28. Bignell M, Lewis MP, Cheong EC, Rhodes M. A prospective, randomized, single-blind trial of 5-mm versus 3-mm ports for laparoscopic cholecystectomy: is smaller better?. Surgical endoscopy. 2013 Oct 1;27(10):3616-21.
29. Bingener J, Skaran P, McConico A, Novotny P, Wettstein P, Sletten DM, Park M, Low P, Sloan J. A double-blinded randomized trial to compare the effectiveness of minimally invasive procedures using patient-reported outcomes. Journal of the American College of Surgeons. 2015 Jul 1;221(1):111-21.
30. ClinicalTrials.gov. *A comparison of single port and four port laparoscopic gallbladder removal CENoMIP*. Bethesda, MD: National Library of Medicine; 2011 [https://clinicaltrials.gov/ct2/show/NCT01489436]
31. Borle FR, Mehra BK, Jain S. Evaluation of pain scores after single-incision and conventional laparoscopic cholecystectomy: A randomized control trial in a rural Indian population. Asian journal of endoscopic surgery. 2014 Jan;7(1):38-42.
32. Brown KM, Moore BT, Sorensen GB, Boettger CH, Tang F, Jones PG, Margolin DJ. Patient-reported outcomes after single-incision versus traditional laparoscopic cholecystectomy: a randomized prospective trial. Surgical endoscopy. 2013 Sep 1;27(9):3108-15.
33. Bucher P, Pugin F, Buchs NC, Ostermann S, Morel P. Randomized clinical trial of laparoendoscopic single-site versus conventional laparoscopic cholecystectomy. British Journal of Surgery. 2011 Dec;98(12):1695-702.
34. Cao ZG, Cai W, Qin MF, Zhao HZ, Yue P, Li Y. Randomized clinical trial of single-incision versus conventional laparoscopic cholecystectomy: short-term operative outcomes. Surgical Laparoscopy Endoscopy & Percutaneous Techniques. 2011 Oct;21(5):311.
35. Chang SK, Wang YL, Shen L, Iyer SG, Shaik AB, Lomanto D. Interim report: A randomized controlled trial comparing postoperative pain in single-incision laparoscopic cholecystectomy and conventional laparoscopic cholecystectomy. Asian journal of endoscopic surgery. 2013 Feb;6(1):14-20.
36. Chang SK, Wang YL, Shen L, Iyer SG, Madhavan K. A randomized controlled trial comparing post-operative pain in single-incision laparoscopic cholecystectomy versus conventional laparoscopic cholecystectomy. World Journal of Surgery. 2015 Apr 1;39(4):897-904.
37. Netherlands trial register. *Single incision, miniport or conventional laparoscopic surgery for uncomplicated symptomatic cholecystolithiasis*. Netherlands: Netherlands Trial Registry; 2011, [https://www.trialregister.nl/trial/2952]
38. Emre TK, Ilhan E, Yakan S, Cengiz F, Senlikci A, Aktürk HE. Single-port laparoscopic cholecystectomy versus the classical four port laparoscopic cholecystectomy: a randomized prospective clinical trial. Minerva chirurgica. 2014 Feb;69(1):1.
39. Herrero EF, Cugat EA, García MD, Camps JL, Porta RC, Carvajal FL, Rodríguez AC. A randomised prospective comparative study between laparoscopic cholecystectomy and single port cholecystectomy in a major outpatient surgery unit. Cirugia espanola. 2012 Dec;90(10):641-6.
40. Guo W, Liu Y, Han W, Liu J, Jin L, Li JS, Zhang ZT. Randomized trial of immediate postoperative pain following single-incision versus traditional laparoscopic cholecystectomy. Chinese medical journal. 2015 Dec 20;128(24):3310.

41. ClinicalTrials.gov. A trial of single port laparoscopic cholecystectomy versus four port laparoscopic cholecystectomy. Bethesda, MD: National Library of Medicine; 2009 [https://clinicaltrials.gov/ct2/show/NCT00892879]

42. Hattori K, Watanabe K, Ogino H, Shimoyana R, Kawachi J, Takeyama H. Prospective comparison between single incision laparoscopic cholecystectomy and needlescopic cholecystectomy. Surgical Endoscopy 2012;26:S249-S430.

43. Hauters P, Auvray S, Cardin JL, Papillon M, Delaby J, Dabrowski A, Framery D, Valverde A, Rubay R, Siriser F, Malvaux P. Comparison between single-incision and conventional laparoscopic cholecystectomy: a prospective trial of the Club Coelio. Surgical endoscopy. 2013 May 1;27(5):1689-94.

44. Jørgensen LN, Rosenberg J, Al-Tayar H, Assaadzadeh S, Helgstrand F, Bisgaard T. Randomized clinical trial of single-versus multi-incision laparoscopic cholecystectomy. British Journal of Surgery. 2014 Mar;101(4):347-55.

45. Khorgami Z, Shoar S, Anbara T, Soroush A, Nasiri S, Movafegh A, Aminian A. A randomized clinical trial comparing 4-port, 3-port, and single-incision laparoscopic cholecystectomy. Journal of investigative surgery. 2014 Jun 1;27(3):147-54.

46. Lai EC, Yang GP, Tang CN, Yih PC, Chan OC, Li MK. Prospective randomized comparative study of single incision laparoscopic cholecystectomy versus conventional four-port laparoscopic cholecystectomy. The American Journal of Surgery. 2011 Sep 1;202(3):254-8.

47. Leung D, Yetasook AK, Carbray J, Butt Z, Hoeger Y, Denham W, Barrera E, Ujiki MB. Single-incision surgery has higher cost with equivalent pain and quality-of-life scores compared with multiple-incision laparoscopic cholecystectomy: a prospective randomized blinded comparison. Journal of the American College of Surgeons. 2012 Nov 1;215(5):702-8.

48. Lirici MM, Califano AD, Angelini P, Corcione F. Laparo-endoscopic single site cholecystectomy versus standard laparoscopic cholecystectomy: results of a pilot randomized trial. The American Journal of Surgery. 2011 Jul 1;202(1):45-52.

49. Luna RA, Nogueira DB, Varela PS, Neto ED, Norton MJ, Luciana do Carmo BR, Peixoto AM, De Mendonça YL, Bendet I, Fiorelli RA, Dolan JP. A prospective, randomized comparison of pain, inflammatory response, and short-term outcomes between single port and laparoscopic cholecystectomy. Surgical endoscopy. 2013 Apr 1;27(4):1254-9.

50. Lurje G, Raptis DA, Steinemann DC, Amygdalos I, Kambakamba P, Petrowsky H, Lesurtel M, Zehnder A, Wyss R, Clavien PA, Breitenstein S. Cosmesis and body image in patients undergoing single-port versus conventional laparoscopic cholecystectomy: a multicenter double-blinded randomized controlled trial (SPOCC-trial). Annals of surgery. 2015 Nov 1;262(5):728-35.

51. Ma J, Cassera MA, Spaun GO, Hammill CW, Hansen PD, Aliabadi-Wahle S. Randomized controlled trial comparing single-port laparoscopic cholecystectomy and four-port laparoscopic cholecystectomy. Annals of surgery. 2011 Jul 1;254(1):22-7.

52. Madureira FA, Manso JE, Madureira Filho D, Iglesias AC. Inflammation in laparoendoscopic single-site surgery versus laparoscopic cholecystectomy. Surgical innovation. 2014 Jun;21(3):263-8.

53. Marks JM, Phillips MS, Tacchino R, Roberts K, Onders R, DeNoto G, Gecelter G, Rubach E, Rivas H, Islam A, Soper N. Single-incision laparoscopic cholecystectomy is associated with improved cosmesis scoring at the cost of significantly higher hernia rates: 1-year results of a prospective randomized, multicenter, single-blinded trial of traditional multiport laparoscopic cholecystectomy vs single-incision laparoscopic cholecystectomy. Journal of the American College of Surgeons. 2013 Jun 1;216(6):1037-47.

54. McGregor CG, Sodergren MH, Aslanyan A, Wright VJ, Purkayastha S, Darzi A, Paraskeva P. Evaluating systemic stress response in single port vs. multi-port laparoscopic cholecystectomy. Journal of Gastrointestinal Surgery. 2011 Apr 1;15(4):614-22.

55. Mehmood ZA, Subhan AN, Ali NA, Rasul SH, Iqbal MA, Khan RA, Karim MT, Shafqutallah SM, Baig MS. Four port versus single incision laparoscopic cholecystectomy. Journal of Surgery Pakistan (International). 2010 Jul;15(3):122.

56. Moran M, Gundogdu E, Dizen H, Akgun AE, Ozmen MM. Single vs two vs three vs four incision laparoscopic cholecystectomy: a randomized controlled trial. Surgical Endoscopy 2011;25:S1-S53.

57. ClinicalTrials.gov. *EndoCone single port versus conventional multi-port laparoscopic approach*. Bethesda, MD: National Library of Medicine; 2012 [https://clinicaltrials.gov/ct2/show/NCT01709877]

58. Ospanov OB, Dildabekov GB. Single-site laparoscopic cholecystectomy using a multiple transumbilical port. Surgical Endoscopy 2012;26:S53-S140.
59. Pan MX, Jiang ZS, Cheng Y, Xu XP, Zhang Z, Qin JS, He GL, Xu TC, Zhou CJ, Liu HY, Gao Y. Single-incision vs three-port laparoscopic cholecystectomy: prospective randomized study. World journal of gastroenterology: WJG. 2013 Jan 21;19(3):394.
60. Pappas-Gogos G, Tellis CC, Trypsianis G, Tsimogiannis KE, Tsimoyiannis EC, Simopoulos CE, Pitiakoudis M, Tselepis AD. Oxidative stress in multi-port and single-port cholecystectomy. journal of surgical research. 2015 Mar 1;194(1):101-6.
61. Pathania BS. A comparative clinical evaluation of single incision laparoscopic cholecystectomy with single and multiport access versus conventional multi-port laparoscopic cholecystectomy. European Surgical Research 2013;50(Suppl 1):1-162.
62. Rašić Ž, Schwarz D, Adam Nesek V, Zoričić I, Sever M, Rašić D, Lojo N. Single incision laparoscopic cholecystectomy–a new advantage of gallbladder surgery. Collegium antropologicum. 2010 Jun 30;34(2):595-8.
63. Sasaki A, Ogawa M, Tono C, Obara S, Hosoi N, Wakabayashi G. Single-port versus multiport laparoscopic cholecystectomy: a prospective randomized clinical trial. Surgical Laparoscopy Endoscopy & Percutaneous Techniques. 2012 Oct 1;22(5):396-9.
64. Sinan H, Demirbas S, Ozer MT, Sucullu I, Akyol M. Single-incision laparoscopic cholecystectomy versus laparoscopic cholecystectomy: a prospective randomized study. Surgical Laparoscopy Endoscopy & Percutaneous Techniques. 2012 Feb 1;22(1):12-6.
65. IRCT.ir. *The comparison between result of single port laparoscopic cholecystectomy with four ports cholecystectomy*. Tehran, Iran: IRCT; 2011 [https://www.irct.ir/trial/6802]
66. Sulu B, Yildiz BD, Ilingi ED, Gunerhan Y, Cakmur H, Anuk T, Yildiz B, Koksal N. Single Port vs. Four Port Cholecystectomy--Randomized Trial on Quality of Life. Advances in clinical and experimental medicine: official organ Wroclaw Medical University. 2015;24(3):469-73.
67. Tono C. Single-port versus multi-port laparoscopic cholecystectomy: a randomised clinical trial. Surgical Endoscopy 2012;26:S53-S140.
68. Ugurlu MU, Oruc MT. Comparison of single-incision laparoscopic cholecystectomy with standard laparoscopic approach. Surgical Endoscopy 2013;27:S304-S503.
69. ClinicalTrials.gov. *A randomised comparison betweennsingle incision laparoscopic cholecystectomy and standard laparoscopic cholecystectomy*. Bethesda, MD: National Library of Medicine; 2010 [https://clinicaltrials.gov/ct2/show/ NCT01094379]
70. Vilallonga R, Barbaros U, Sümer A, Demirel T, Fort JM, González O, Rodriguez N, Carrasco MA. Single-port transumbilical laparoscopic cholecystectomy: a prospective randomised comparison of clinical results of 140 cases. Journal of minimal access surgery. 2012 Jul;8(3):74.
71. Zapf M, Yetasook A, Leung D, Salabat R, Denham W, Barrera E, Butt Z, Carbray J, Du H, Wang CE, Ujiki M. Single-incision results in similar pain and quality of life scores compared with multi-incision laparoscopic cholecystectomy: a blinded prospective randomized trial of 100 patients. Surgery. 2013 Oct 1;154(4):662-71.
72. Agrawal R, Sood KC, Agarwal B. Evaluation of early versus delayed laparoscopic cholecystectomy in acute cholecystitis. Surgery Research and Practice. 2015 Feb 3;2015.
73. Faizi KS, Ahmed I, Ahmad H. Comparison of early versus delayed laparoscopic cholecystectomy: choosing the best. Pak J Medical Health Sci. 2013;7:212-5.
74. Gul R, Dar RA, Sheikh RA, Salroo NA, Matoo AR, Wani SH. Comparison of early and delayed laparoscopic cholecystectomy for acute cholecystitis: experience from a single center. North American journal of medical sciences. 2013 Jul;5(7):414.
75. Gutt CN, Encke J, Köninger J, Harnoss JC, Weigand K, Kipfmüller K, Schunter O, Götze T, Golling MT, Menges M, Klar E. Acute cholecystitis: early versus delayed cholecystectomy, a multicenter randomized trial (ACDC study, NCT00447304). Annals of surgery. 2013 Sep 1;258(3):385-93.
76. Johansson M, Thune A, Blomqvist A, Nelvin L, Lundell L. Management of acute cholecystitis in the laparoscopic era: results of a prospective, randomized clinical trial. Journal of gastrointestinal surgery. 2003 Jul 1;7(5):642-5.
77. Johansson M, Thune A, Nelvin L, Stiernstam M, Westman B, Lundell L. Randomized clinical trial of open versus laparoscopic cholecystectomy in the treatment of acute cholecystitis. British journal of surgery. 2005 Jan 1;92(1):44-9.

78. Kolla SB, Aggarwal S, Kumar A, Kumar R, Chumber S, Parshad R, Seenu V. Early versus delayed laparoscopic cholecystectomy for acute cholecystitis: a prospective randomized trial. Surgical Endoscopy and Other Interventional Techniques. 2004 Sep 1;18(9):1323-7.
79. Lai PB, Kwong KH, Leung KL, Kwok SP, Wchan AC, Chung SC, Lau WY. Randomized trial of early versus delayed laparoscopic cholecystectomy for acute cholecystitis. British journal of surgery. 1998 Jun 1;85(6):764-7.
80. Lo CM, Liu CL, Fan ST, Lai EC, Wong J. Prospective randomized study of early versus delayed laparoscopic cholecystectomy for acute cholecystitis. Annals of surgery. 1998 Apr;227(4):461.
81. Macafee DA, Humes DJ, Bouliotis G, Beckingham IJ, Whynes DK, Lobo DN. Prospective randomized trial using cost–utility analysis of early versus delayed laparoscopic cholecystectomy for acute gallbladder disease. British journal of surgery. 2009 Sep;96(9):1031-40.
82. Mare LD, Saadi A, Roulin D, Demartines N, Halkic N. Delayed versus early laparoscopic cholecystectomy for acute cholecystitis: a prospective randomized study. HBP 2012;14(Suppl 2):107-287.
83. Özkardeş AB, Tokaç M, Dumlu EG, Bozkurt B, Çiftçi AB, Yetişir F, Kılıç M. Early versus delayed laparoscopic cholecystectomy for acute cholecystitis: a prospective, randomized study. International surgery. 2014;99(1):56-61.
84. Saber A, Hokkam EN. Operative outcome and patient satisfaction in early and delayed laparoscopic cholecystectomy for acute cholecystitis. Minimally invasive surgery. 2014 Jan 1;2014.
85. Salman B, Yüksel O, Irkörücü O, Akyürek N, Tezcaner T, Doğan İ, Erdem Ö, Tatlicioğlu E. Urgent laparoscopic cholecystectomy is the best management for biliary colic. Digestive surgery. 2005;22(1-2):95-9.
86. Verma S, Agarwal PN, Bali RS, Singh R, Talwar N. Early versus delayed laparoscopic cholecystectomy for acute cholecystitis: a prospective randomized trial. International Scholarly Research Notices. 2013;2013.
87. Yadav RP, Adhikary S, Agrawal CS, Bhattarai B, Gupta RK, Ghimire A. A comparative study of early vs. delayed laparoscopic cholecystectomy in acute cholecystitis. Kathmandu University medical journal. 2009;7(1):16-20.
88. Alhashemi M, Almahroos M, Fiore JF, Kaneva P, Gutierrez JM, Neville A, Vassiliou MC, Fried GM, Feldman LS. Impact of miniport laparoscopic cholecystectomy versus standard port laparoscopic cholecystectomy on recovery of physical activity: a randomized trial. Surgical endoscopy. 2017 May 1;31(5):2299-309.
89. Alponat A, Çubukçu A, Gönüllü N, Cantürk Z, Öbay O. Is minisite cholecystectomy less traumatic? Prospective randomized study comparing minisite and conventional laparoscopic cholecystectomies. World journal of surgery. 2002 Dec 1;26(12):1437-40.
90. de Carvalho LF, Fierens K, Kint M. Mini-laparoscopic versus conventional laparoscopic cholecystectomy: a randomized controlled trial. Journal of Laparoendoscopic & Advanced Surgical Techniques. 2013 Feb 1;23(2):109-16.
91. Gupta A, Shrivastava UK, Kumar P, Burman D. Minilaparoscopic versus laparoscopic cholecystectomy: a randomised controlled trial. Tropical gastroenterology: official journal of the Digestive Diseases Foundation. 2005 Jul 1;26(3):149-51.
92. Hsieh CH. Early minilaparoscopic cholecystectomy in patients with acute cholecystitis. The American journal of surgery. 2003 Apr 1;185(4):344-8.
93. Huang MT, Wang W, Wei PL, Chen RJ, Lee WJ. Minilaparoscopic and laparoscopic cholecystectomy: a comparative study. Archives of Surgery. 2003 Sep 1;138(9):1017-23.
94. Leggett PL, Churchman-Winn R, Miller G. Minimizing ports to improve laparoscopic cholecystectomy. Surgical endoscopy. 2000 Jan 1;14(1):32-6.
95. Novitsky YW, Kercher KW, Czerniach DR, Kaban GK, Khera S, Gallagher-Dorval KA, Callery MP, Litwin DE, Kelly JJ. Advantages of mini-laparoscopic vs conventional laparoscopic cholecystectomy: results of a prospective randomized trial. Archives of Surgery. 2005 Dec 1;140(12):1178-83.
96. Saad S, Strassel V, Sauerland S. Randomized clinical trial of single-port, minilaparoscopic and conventional laparoscopic cholecystectomy. British Journal of Surgery. 2013 Feb;100(3):339-49.
97. Velázquez-Mendoza JD, Villagrán-Murillo FJ, González-Ojeda A. Minilaparotomy vs. laparoscopic cholecystectomy: results of a randomized clinical trial. Cirugía y Cirujanos. 2012;80(2):115-21.
98. Ahmed W, Gaber A, Elmoghazy W, Elsharkawy K. Mini-laparotomy cholecystectomy versus laparoscopic cholecystectomy: a randomized controlled trial. Surgical Endoscopy 2016;30:S63-S156.
99. Aspinen S, Harju J, Juvonen P, Kokki H, Remes V, Scheinin T, Eskelinen M. A prospective, randomized multicenter study comparing conventional laparoscopic cholecystectomy versus minilaparotomy

cholecystectomy with ultrasonic dissection as day surgery procedure—1-year outcome. Scandinavian journal of gastroenterology. 2014 Nov 1;49(11):1336-42.
100. Aspinen A, Harju J, Juvonen P, Kokki H, Remes V,Scheinin, Eskelinen M. Minilaparotomy cholecystectomy with ultrasonic dissection versus conventional laparoscopic cholecystectomy-1 year outcome. Surgical Endoscopy 2015;29:S75-S252.
101. Harju J. Comparison of the quality of life after minilaparotomy cholecystectomy versus laparoscopic cholecystectomy: a prospective randomized study. Energy. 2007 Mar 1;72(69):0-399.
102. Harju J, Juvonen P, Kokki H, Remes V, Scheinin T, Eskelinen M. Minilaparotomy cholecystectomy with ultrasonic dissection versus conventional laparoscopic cholecystectomy: a randomized multicenter study. Scandinavian journal of gastroenterology. 2013 Nov 1;48(11):1317-23.
103. Harju J, Aspinen S, Juvonen P, Kokki H, Eskelinen M. Ten-year outcome after minilaparotomy versus laparoscopic cholecystectomy: a prospective randomised trial. Surgical endoscopy. 2013 Jul 1;27(7):2512-6.
104. Dirksen CD, Schmitz RF, Hans KM, Nieman FH, Hoogenboom LJ, Go PM. Ambulatory laparoscopic cholecystectomy is as effective as hospitalization and from a social perspective less expensive: a randomized study. Nederlands tijdschrift voor geneeskunde. 2001 Dec 15;145(50):2434.
105. Hollington P, Toogood GJ, Padbury RT. A prospective randomized trial of day-stay only versus overnight-stay laparoscopic cholecystectomy. Australian and New Zealand Journal of Surgery. 1999 Dec;69(12):841-3.
106. Johansson M, Thune A, Nelvin L, Lundell L. Randomized clinical trial of day-care versus overnight-stay laparoscopic cholecystectomy. British Journal of Surgery. 2006 Jan 1;93(1):40-5.
107. Young J, O'Connell B. Recovery following laparoscopic cholecystectomy in either a 23 hour or an 8 hour facility. Journal of Quality in Clinical Practice. 2001 Mar;21(1-2):2-7.
108. Plaisier PW, Van der Hul RL, Nijs HG, Den Toom R, Terpstra OT, Bruining HA. The course of biliary and gastrointestinal symptoms after treatment of uncomplicated symptomatic gallstones: results of a randomized study comparing extracorporeal shock wave lithotripsy with conventional cholecystectomy. American Journal of Gastroenterology. 1994 May 1;89(5).
109. Vethrus M, Søreide O, Solhaug JH. Symptomatic, non-complicated gallbladder stone disease. Operation or observation. Scand J Gastroenterol. 2002;37:834-9.
110. Vetrhus M, Søreide O, Nesvik I, Søndenaa K. Acute cholecystitis: delayed surgery or observation. A randomized clinical trial. Scandinavian journal of gastroenterology. 2003 Sep;38(9):985-90.
111. Russell, M. L., Preshaw, R. M., Brant, R. F., Bultz, B. D., & Page, S. A. (1996). Disease-specific quality of life: the Gallstone Impact Checklist. *Clinical and Investigative Medicine. Médecine Clinique Et Experimentale*, *19*(6), 453-460.
112. Urbach, D., Harnish, J., McIlroy, J., & Streiner, D. (2006). A measure of quality of life after abdominal surgery. *Quality of Life Research*, *15*(6), 1053-1061.
113. Chen TY, Landmann MG, Potter JC, et al. Questionnaire to aid priority and outcomes assessment in gallstone disease. ANZ J Surg 2006;76:569-74. 23.
114. Eypasch, E., ood-Dauphin e, S., Williams, J. I., Ure, B., Neugebauer, E., & Troidl, H. (1993). The Gastrointestinal Quality of Life Index. A clinical index for measuring patient status in gastroenterologic surgery. *Der Chirurg,64*(4), 264-74.
115. Lindseth GN, Denny DL. Patients' experiences with cholecystitis and a cholecystectomy. Gastroenterol Nurs. 2014 Nov-Dec;37(6):407-14
116. Kleinbeck SV, Hoffart N. Outpatient recovery after laparoscopic cholecystectomy. AORN J. 1994 Sep;60(3):394, 397-8, 401-2.
117. Psaila J, Agrawal S, Fountain U, Whitfield T, Murgatroyd B, Dunsire MF, Gonzalez JG, Patel AG. Day-surgery laparoscopic cholecystectomy: factors influencing same-day discharge. World J Surg. 2008 Jan;32(1):76-81
118. Peron A, Schliemann AL, Almeida FA. Understanding the reasons for the refusal of cholecystectomy in patients with cholelithiasis: how to help them in their decision? Arq Bras Cir Dig. 2014 Apr-Jun;27(2):114-9.
119. McKneally MF, Ignagni E, Martin DK, D'Cruz J. The leap to trust: perspective of cholecystectomy patients on informed decision making and consent. J Am Coll Surg. 2004 Jul;199(1):51-7.
120. Barthelsson C, Lützén K, Anderberg B, Nordström G. Patients' experiences of laparoscopic cholecystectomy in day surgery. J Clin Nurs. 2003 Mar;12(2):253-9.

For peer review only - http://bmjopen.bmj.com/site/about/guidelines.xhtml

121. Keus F, de Vries J, Gooszen HG, van Laarhoven CJ. Assessing factors influencing return back to work after cholecystectomy: a qualitative research. BMC Gastroenterol. 2010 Jan 27;10:12
122. Ros E, Zambon D. Postcholecystectomy symptoms. A prospective study of gall stone patients before and two years after surgery. Gut. 1987 Nov;28(11):1500-4.
123. Alkhaffaf B, Blazeby JM, Williamson PR, Bruce IA, Glenny AM. Reporting of outcomes in gastric cancer surgery trials: a systematic review. BMJ Open. 2018 Oct 17;8(10):e021796.
124. Young AE, Davies A, Bland S, Brookes S, Blazeby JM. Systematic review of clinical outcome reporting in randomised controlled trials of burn care. BMJ Open. 2019 Feb 15;9(2):e025135.
125. Daliya P, Gemmill EH, Lobo DN, Parsons SL. A systematic review of patient reported outcome measures (PROMs) and quality of life reporting in patients undergoing laparoscopic cholecystectomy. Hepatobiliary Surg Nutr. 2019 Jun;8(3):228-245
126. Alexander HC, Nguyen CH, Moore MR, Bartlett AS, Hannam JA, Poole GH, Merry AF. Measurement of patient-reported outcomes after laparoscopic cholecystectomy: a systematic review. Surg Endosc. 2019 Jul;33(7):2061-2071
127. Klokker, L., Berthelsen, DB.; Woodworth, T. et al.Identifying Possible Outcome Domains from Existing Outcome Measures to Inform an OMERACT Core Domain Set for Safety in Rheumatology Trials By: Conference: Outcome Measures in Rheumatology Conference (OMERACT) Location: Terrigal, AUSTRALIA Date: MAY 15-18, 2018
128. Duncan, R.; Essat, M.; Jones, G.; et al. Systematic review and qualitative evidence synthesis of patient-reported outcome measures for abdominal aortic aneurysm. B :
129. Shilling, V., Matthews, L., Jenkins, V. Patient-reported outcome measures for cancer caregivers: a systematic review. Quality of Life Resarrch. 2016. 25(8):1859-1876.
130. Harman NL, Wilding JPH, Curry D on behalf of the SCORE-IT Study Team*, et al.* Selecting Core Outcomes for Randomised Effectiveness trials In Type 2 diabetes (SCORE-IT): a patient and healthcare professional consensus on a core outcome set for type 2 diabetes. *BMJ Open Diabetes Research and Care* 2019;7:e000700.
131. Blencowe NS, Strong S, McNair AG, Brookes ST, Crosby T, Griffin SM, Blazeby JM. Reporting of short-term clinical outcomes after esophagectomy: a systematic review. Ann Surg. 2012 Apr;255(4):658-66.
132. Whistance RN, Forsythe RO, McNair AG, Brookes ST, Avery KN, Pullyblank AM, Sylvester PA, Jayne DG, Jones JE, Brown J, Coleman MG, Dutton SJ, Hackett R, Huxtable R, Kennedy RH, Morton D, Oliver A, Russell A, Thomas MG, Blazeby JM; Core Outcomes and iNformation SEts iN SUrgical Studies - ColoRectal Cancer Working Group. A systematic review of outcome reporting in colorectal cancer surgery. Colorectal Dis. 2013;15(10):e548-60.

Authors contributions

Conceptualisation CR

Methodology MC, RN, JB, IA, MBr, CR, KG

Investigation MC, RN, JB, MB, BC, KG

Formal Analysis MC, RN, JB, IA, MB, CR, KG

Writing – Original draft MC, RN, KG

Writing – Review and Editing MC, RN, JB, IA, MB, KI, CR, KG

Visualisation MC, RN, KG

Supervision KG

Project Administration MC, RN, KI, KG

Funding Acquisition JB, IA, MBr, BC, CR, KG

Funding statement

This work was supported by the National Institute for Health Research (NIHR) Health Technology Assessment (HTA) Programme grant [14/192/71]. The work was also supported by an NHS Grampian Endowment grant [16/11/006]. KG held a Medical Research Council UK Methodology Fellowship during the delivery of this project [MR/L01193X/1]. The Health Services Research Unit, Institute of Applied Health Sciences (University of Aberdeen), is core-funded by the Chief Scientist Office of the Scottish Government Health and Social Care Directorates. The funders had no involvement in study design, collection, analysis and interpretation of data, reporting or the decision to publish.

Competing interests

JB is a member of the COMET Initiative Management Group. No others to declare.

Provenance and peer review

Not commissioned; externally peer reviewed.

Data sharing statement

No further data are available.

For peer review only

Table 1: Summary characteristics and demographics of included studies

Characteristic	Quantitative review	Qualitative review
Number of study participants	(n=119 studies)	n=8 studies
Median	75	19.5
Range	14-618	6-100
Total	10757	256
No of males/females	(n=96 studies)	(n=7 studies)
Median	21/53.5	4/16
Range	0-255/15-415	2-15/4-37
Total	2632/6166	43/113
Mean age (years)	(n=14 studies)	-
Median (years)	46.1	-
Range (years)	40-53	19-81
Country	(n=128 studies)	(n=8 studies)
UK	7[a]	1
EEA (excl UK)	44[a]	3
USA	12[a]	1
Other	66	3
No of Centres	(n=129 studies)	(n=8 studies)
Single centre	113	8
Multicentre	16	
Total no of outcomes reported	330	17

[a]One study (Marks 2013) was conducted in the UK, USA and Italy and is included in the count for each country; Number of studies (n=xx studies) in table relates to how many studies reported each relevant characteristic

For peer review only

2

For peer review only

Table 2 Outcomes reported in randomised controlled trials of interventions to treat uncomplicated gallstone disease

Domain	Outcome	Number of times reported
**Physical**	Physical activity postop	1
	Recovery of self-reported physical activity	1
	Time to resume normal physical activity	3
	Time to resume walking	3
	Functional impairment	1
**Role**	Time to everyday life	1
	Return to daily activities	1
	Return to normal function	1
	Return to activities of daily living	1
	Days from surgery to normal activity	3
	Time to return to normal activities	1
	Days to full activity	1
	No of days sick leave	3
	Time away from work	2
	Loss of active days of work	1
	Time to return to work after discharge	1
	Time to return to work	8
	Days to return to school	1
	Convalescence time	1
	Postop regain of functionality	1
	Total recuperation period	1
	Mobilisation	1
**Pain**	Overall pain	2
	Pain	2
	Visual analogue pain score	2
	Site of most severe pain	1
	Umbilical pain	1
	Abdominal pain	9
	Right upper quadrant pain attacks	3

For peer review only - http://bmjopen.bmj.com/site/about/guidelines.xhtml

	Shoulder pain	4
	Admission due to pain	2
	Incisional pain	10
	Whether had pain or other symptoms in incision area/port sites	1
	Patient-reported location of pain at initial postop visit	1
	Postoperative pain	72
	Chronic post surgery pain	1
	Analgesic requirements	58
	Duration of analgesia use	2
	Time to first analgesics	1
	Days of medication required	1
	Analgesia requirement	1
	No of patients needing additional analgesia	1

For peer review only

	Use of peripheral analgesics	1
	Use of centrally acting analgesia	1
	Total pethidine (mg)	1
	Pethidine requirement	1
	Suppository requests	1
**Bowel movements**	Return of bowel function	1
	Change in bowel habit	1
	Diarrhoea or loose stools	2
	Time to resume passing stools	1
**Thirst/dehydration**	Resumption of oral diet within 24h	1
	Resumption of orals	1
	Resumption of oral intake	3
	Normal drinking	1
	Return to liquid feeds	1
	Time to clear liquids	1
**Appetite/eating/taste**	Time to resume eating	4
	Normal eating	1
	Return to solid diet	2
	Time to resume feeding	1
**Fatigue**	Fatigue	2
**Sleep**	Length of night sleep	1
	Awakening during the night	1
**Generic health**	Overall health state	1
	Health status	1
	Satisfaction with life in general	1
	Patient satisfaction	2
	Overall satisfaction	2
	Satisfaction score	1
	Global patient satisfaction	1
	Patient overall satisfaction	1
	Time interval between onset of symptoms and admission to hospital	1
**Dietary habits**	Need to avoid fried/fatty foods after surgery	3
**Social**	Time away from recreational activity	1

For peer review only

	Return to going out (days)	1
**Belching/Bloating/Gas**	Time to resume passing intestinal gases	1
	Flatulence and/or dyspepsia	1
**Vomiting/nausea**	Nausea or vomiting	5
	Vomiting	7
	Nausea	8
**Reflux**	Reflux symptoms	2
	Heartburn or regurgitation	1
**Regurgitation**	Heartburn or regurgitation	1
**Body Image**	Satisfaction with body image	3
	Patient derived body image	1
	Satisfaction with aesthetic result	1
	Score on body image scale	2
	Wound satisfaction	2
	Satisfaction with cosmetic outcome	10
	Incision satisfaction	1

For peer review only

	Patient satisfaction score on surgery and scars	2
	Cosmetic satisfaction of surgical scar	2
	Patient satisfaction score on scars	1
	Cosmetic outcome	13
	Score on cosmetic scale	1
	Cosmesis	5
	Cosmetic score	1
	Cosmetic result	6
	Incisional cosmesis	1
	Incision cosmetic result	1
	Scar evaluation	4
	Appearance of each incision	1
**Sexual function**	Satisfaction in the context of sexual intercourse	1
	Pain in the context of sexual intercourse	1
	Dyspareunia	1
**Generic symptoms**	Quality of life	22
	Whether operation had had any impact on QoL	1
	Morbidity	8
	Overall morbidity following diagnosis	1
	General discomfort	1
	Symptoms	1
	Symptoms during waiting period	2
	Residual abdominal symptoms	3
	Examined for residual symptoms	1
	Gastrointestinal complaints	1
	Failure of conservative treatment	4
	Failure of technique	1
	No of patients recovered	1
	Complication rates during waiting time for elective laparoscopic cholecystectomy	1
	Success rate	2
**Mortality**	Mortality	30
**Intra-operative adverse events**	CBD stones	1
	CBD injury	3

	Biliary leak	1
	Bile leakage	2
	Biliary injury	1
	Bile duct injury	2
	Bile spillage	1
	Bile duct lesions	1
	Haemorrhage	1
	Blood loss	30
	Rate of intraoperative bleeding	1
	Intraoperative bleeding	1
**Intra and post-operative adverse events**	Adverse events	6
	Intra- and postoperative complications	1
	Operative complications	43
	Complications	6
	Other complications	1
	General complications	2

For peer review only

For peer review only - http://bmjopen.bmj.com/site/about/guidelines.xhtml

	Global complications	1
	Major complications	1
	Abdominal wall complications	1
	Port-site complications	1
	Tissue damage	1
	Intra-abdominal collections	1
**Post operative adverse events**	Postoperative complications	66
	Parietal complications after surgery	2
	Postoperative infection rate	1
	Wound infection(s)	7
	Rate of wound infection	1
	Surgical site infection	1
	Incision infection	1
	Postop wound-related infection	1
	Wound condition	1
	Postop wound-related hernia	1
	Port hernia	2
	Postoperative hernia	1
	Incisional hernia occurrence	6
	Trocar herniation	2
	Intra-abdominal adhesions	1
	Satisfaction with surgery in general	1
	Patient satisfaction score on surgery	2
	Satisfaction with operation	5
	Satisfaction with overall procedural result	1
	Patient satisfaction after surgery	1
	Perceived success of operation	1
	Recommends the procedure	3
	Patient preference survey including "willingness to pay for SILC" questionnaire	1
	Procedure believed to have undergone	1
**Service use**	Readmission before elective operation	1
	Operating time	103
	Operative data	2

For peer review only - http://bmjopen.bmj.com/site/about/guidelines.xhtml

	Surgical time	2
	Extensions	1
	Procedure time	1
	System setup time	1
	Performance time	1
	Duration of each operative stage	1
	Learning curve for operating time	1
	Duration of admission	1
	Hospital stay	60
	Postoperative hospital stay	23
	Day surgery	2
	Successful completion of day surgery	3
	Reasons for overnight stay in patients scheduled for day surgery	1
	PACU length of stay	1
	Discharge > 24h postop	1

For peer review only

For peer review only - http://bmjopen.bmj.com/site/about/guidelines.xhtml

	Discharge from hospital	1
	Discharge from hospital 20-24h postop	1
	Time until discharge	1
	Time from operation to discharge	1
	Grade of surgeon	1
	Grade of operating surgeon	1
	Resident's participation	1
	Need to contact hospital or other health care providers after discharge	2
	Ambulatory rate	1
	No of patients requiring readmission to hospital	5
	Readmission	3
	Causes of hospitalisation	2
	No of patients requiring re-intervention	2
	Additional procedures	1
	Reoperation	1
	Reintervention required	1
	Revision surgery	1
	Conversion to open surgery	71
	Conversion of 5mm to 10mm port	2
	Modification of operative technique	4
	Conversion to LC	16
	Conversion to LESS procedure	1
	Conversion to other laparoscopic approach	4
	Conversion	2
	Conversion from SILC to 4PLC	2
	Rate of cholecystectomy	2
**Cost-effectiveness**	Cost of operation	6
	Cost analysis	1
	Procedural cost	1
	Total encounter cost	1
	Hospital cost	5
	Hospital charges	1
	Total cost	1

For peer review only

	Costs	1
	Charge data	1
	Cost-effectiveness ratio	1
	Economic analysis of early versus conventional management for newly diagnosed GB disease	1
**Process (operation or trial)**	Pulmonary function	5
	Spirometric indices	1
	Total Co2 insufflation (litres)	1
	Surgical stress response	2
	No of cannulas used	1
	Pressure of the pneumo peritoneum	1
	Heart rate variability	1
	Serum cytokines	1
	Serum interleukin-6	3
	Serum C-reactive protein	3
	Blood count	1

For peer review only

For peer review only - http://bmjopen.bmj.com/site/about/guidelines.xhtml

	Liver function tests	1
	Alpha-defensins expression	1
	hsCRP values	1
	CRP values	1
	Electroconductivity of representative dermatones	1
	Central & peripheral temperature	1
	Blood examination	1
	Histological findings	1
	Histopathologic findings	1
	Histopathological diagnosis	1
	Occlusive bandages in place	1
	General anaesthesia	1
	Anaesthetic time	1
	Amount of bupivacaine used	1
	Length of skin incision	9
	Extra skin incisions required	1
	Aponeurosis wound size	1
	Wound length	3
	Scar length	2
	Intraoperative cholangiography performed	3
	Intraoperative technical performance	1
	Intraoperative diagnosis	1
	Intraoperative findings	3
	Postop metabolic & hormonal levels	1
	Postoperative level of 8-epiPGF2α	1
	Postoperative level of uric acid	1
	Postoperative forced expiratory volume in 1 second	1
	Change of antibiotic therapy due to nonresponse or to intolerance of moxifloxacin	1
	Use of rescue therapy with 125mg lysine clonixinate tablets	1
	Time of surgical dissection	1
	Time of closure	1
	Detailed surgical time course	1

For peer review only

	Rate of operative error	1
	Technical problems	2
	No of admissions between 8am & 5pm	1
	Admission on Monday to Thursday	1
	Difficulty of case	2
	Operative difficulty	1
	Surgical difficulty	1
	Difficulty of dissection	1
	Difficulty (impaired) of exposure	1
	Prediction of LC difficulty	1
	Surgeon's perceptions of difficulties during operation	1
	Potential for increased surgical risk to the patient	1
	Critical view of safety	1
	Assessment of surgical handling	1
	Feasibility and safety of SILC vs 4PLC	1

For peer review only

	Ultrasonography findings	1
	No of stones	1
	Size of largest stone	1
	Rate of GB rupture	1
	GB wall thickness	1
	Time to retrieve GB	1
	Number & type of instruments	1
	Use of extra umbilical rescue device 2.3mm mini-LC instrument	1
	Insertion points	1
	Number of incisions	1
	Port sizes	1
	Additional ports required	9
	Port enlargement for GB removal	1
	Additional trocars used	2
	Fourth trocar added	2
	Trocar use	1
	Placement of any additional laparoscopic ports other than the SILS port	1
	Time required for insertion of SILS port system compared to 4 standard ports	2
	Success rate (3-port vs 4-port LC)	2
	Need to amplify extraction port	2
	No of ports used	1
	Reason for port placement	1
	Time of trocar introduction	1
	Use of Keith needle	1
	Successful completion of needlescopic operation	1
	No of drains placed	1
	Requirement of drains	1
	No of manual camera corrections	1
	No of cleaning of the optics	1
	Ability to achieve optimal focus on the operative site during the procedure	1

For peer review only

	Positioning accuracy of image	1
	No of clearing camera	1
	Time of adjusting operative field	1
	Surgeon's comfort	1
	Comfort of instrument positioners	1
	No of actions in positioning of laparoscope	1
	Feasibility of surgical procedures using only the camera robot	1
	Commands misunderstood	1
	No of commands issued by the surgeon	1
	Sum of operative actions	1
	No of dissection actions	1
	No of grasping actions	1
	Time from admission to ultrasonography	1
	Time from ultrasonography to recruitment	1
	Time from recruitment to operation	1

For peer review only

For peer review only - http://bmjopen.bmj.com/site/about/guidelines.xhtml

	Time from recruitment to discharge	1
	Completion of randomised treatment	2
	Gallstone-associated events after randomisation	2

For peer review only

For peer review only

For peer review only - http://bmjopen.bmj.com/site/about/guidelines.xhtml

Table 3. Inclusion of domains across included PROMs ranked by frequency of domains.

Domain (n=22)	No. items per domain	ASIS[112]	CSQ[113]	GIC[111]	GIQLI[114]	*TOTAL*
Emotional	17	✓	✓	✓	✓	4
Pain	17	✓	✓	✓	✓	4
Role	11	✓	✓			2
Bowel movements	8	✓			✓	2
Belching/Bloating/Gas	7			✓	✓	2
Appetite/Eating/Taste	6	✓		✓	✓	3
Dietary habits	6		✓	✓		2
Physical	6	✓			✓	2
Fatigue	5	✓	✓	✓	✓	4
Social	4		✓	✓	✓	3
Sleep	3	✓			✓	2
Stomach problems	3		✓	✓		2
Generic health	2		✓		✓	2
Reflux	2			✓		1
Vomiting/nausea	2			✓		1
Body Image	1				✓	1
Cognitive	1	✓				1
Dysphagia/swallowing	1				✓	1
Regurgitation	1				✓	1
Service use	1			✓		1
Sexual function	1				✓	1
Thirst/dehydration	1	✓				1
TOTAL	106	*10*	*8*	*11*	*14*	

For peer review only

11

For peer review only

Table 4. Outcomes reported in PROMs, qualitative evidence synthesis and primary qualitative research

Outcome	PROMs	Evidence synthesis	Primary research
Emotional	✓	✓	✓
Pain	✓	✓	✓
Role	✓	✓	
Bowel movements	✓	✓	✓
Belching/Bloating/Gas	✓	✓	✓
Appetite/Eating/Taste	✓		
Dietary habits	✓	✓	✓
Physical	✓		
Fatigue	✓	✓	✓
Social	✓	✓	✓
Sleep	✓	✓	✓
Stomach problems	✓	✓	✓
Generic health	✓		
Reflux	✓	✓	✓
Vomiting/nausea	✓	✓	✓
Body Image	✓		
Cognitive	✓		
Dysphagia/swallowing	✓		
Regurgitation	✓		
Service use	✓		✓

For peer review only

**Sexual function**	✓		
**Thirst/dehydration**	✓		
**Dizziness**		✓	
**Fainting**		✓	
**Prevention of additional disease**		✓	
**Trust**		✓	
**Weight**		✓	
**Cough**			✓
**Mortality**			✓
**Problems with breathing**			✓
**TOTAL**	22	17	17
**TOTAL INDIVIDUAL OUTCOMES**		**30**	

For peer review only

Table 5. Outcomes Map for uncomplicated gallstone disease

Outcome Domains	Outcome Types	Definitions	Contributing evidence source
Physical	Physical activity	Activities such as walking, running, swimming, cycling, physical labour, climbing stairs, gardening etc	1, 2
	Exercise	Being able to do activities requiring physical effort, carried out to sustain or improve health and fitness (strength and endurance)	3
Role	No. of days sick leave	Length of time off work after the operation in days	1,2,3
	Time to everyday life	Length of time taken to return to usual everyday activities	1,2,3,4
	Impact on others	Impact of your gallstone condition or your gallstone surgery on relationships with people surrounding you	2,3,4
Pain	Overall pain	Overall pain	1,2,3,4
	Abdominal pain	General pain occurring at rest and/or when coughing, originating in the abdominal area	1,2,3,4
	Umbilical pain	Pain around the belly button scar (this is where the main port is that removed the stones)	1
	Shoulder pain	Pain relating to or affecting the right shoulder region	1, 2
Bowel movements	Diarrhoea	Watery stools, loose bowel motion	1,2,3,4
	Constipation	Difficulty passing stool	2,3,4
Thirst/dehydration	Resumption of orals	Starting to eat and drink after treatment	1, 2

For peer review only

Appetite/eating/taste	Time to resume eating	Length of time taken to return to oral food intake	1,2,3,4
Fatigue	Fatigue	Feeling physically or mentally tired or lacking in energy	1,2,3,4
Sleep	Length of night sleep	Length of night's sleep	1,2,3,4
Cognitive	Difficulty concentrating	Inability to focus attention on one task or problem	2
Emotional	Anxiety	A feeling of worry, nervousness or unease	2,3,4
	Distress	A feeling of extreme anxiety, stress or anguish	2,3,4
	Trust	belief in the reliability, truth, or ability of someone or something	3
Generic health	Quality of life	How well you feel physically and emotionally because of a combination of:<br>Your gallstones<br>The prospect of treatment<br>The result of treatment (Treatment might include surgery or painkillers)	1,2,3,4

For peer review only

	Overall health state	Overall state of your physical and mental condition	1
	Overall satisfaction	The degree to which expectations or needs have been fulfilled	1
Dietary habits	Food intolerance	A physical adverse reaction by the body to certain foods	1, 4
Social	Time away from recreational activities	Time spent away from enjoyable activities as a result of your gallstone condition or gallstone surgery	1,2,3,4
Belching/bloating/gas	Flatulence	Belching, farting, bloating or gas	1,2,3,4
	Bloating	Abdominal swelling as a result of excess fluid or gas	2,3,4
	Abdominal discomfort	Pain or discomfort in the stomach area	2,3,4
Vomiting/nausea	Vomiting	Being sick	1,2,3,4
	Nausea	Feeling sick	1,2,3,4
Reflux	Heartburn	A form of indigestion that presents as a burning sensation in the chest, caused by acid reflux	1,2,3,4
Body image	Satisfaction with body image	A feeling of satisfaction with your own physical appearance	1, 2
	Satisfaction with cosmetic outcome	The extent to which you are content with the cosmetic results of gallstone surgery	1, 2
Sexual function	Satisfaction in the context of sexual intercourse	The extent to which you are satisfied with experiences of sexual intercourse in relation to your gallstone condition or your gallstone surgery	1, 2
	Pain in the context of	The extent to which you are experiencing pain during or after sexual intercourse in relation	1, 2

For peer review only

	sexual intercourse	to your gallstone condition or your gallstone surgery	
Regurgitation	Regurgitation	Bringing swallowed food back up to the mouth	1, 2
Dysphagia/swallowing	Trouble swallowing food	Problems swallowing food	2
Generic symptoms	General discomfort	An unpleasant feeling and/or low-level pain which is hard to define	1,2,3,4
	Residual symptoms	Continuing to have symptoms (such as pain, bloating, etc) after removal of the gallbladder	1,2,3,4
	Dizziness	Feeling light headed or dizzy	3
	Fainting	Fainting (short-term loss of consciousness)	3
Mortality	Mortality	Death from any cause	1,4
Intra-operative adverse events	Common bile duct stones	Stones in the common bile duct	1

For peer review only

	Common bile duct injury	During surgery the common bile duct is damaged	1
	Biliary leak	Liver produces bile which is stored in the gallbladder (see diagram). If this is damaged the bile can leak and cause complications.	1
	Haemorrhage	Bleeding or the abnormal flow of blood; the release of blood from a ruptured blood vessel	1
Intra and post-operative adverse events	Intra-abdominal collections	After surgery any type of fluid collecting in the abdomen.	1
Post operative adverse events	Hernia occurrence	internal hernia – displacement of an organ within the abdomen through a potential defect.	1
	Port-site complications	Complications such as infection, hernia, pain or bleeding at or within the 'keyholes' characteristic of keyhole surgery	1
	Wound infections	An infection at the wound site	1
	Patient perceived success of operation	How patient perceive the success of the operation	1, 3
Service use	Hospital stay	Length of time spent in the hospital from admission to discharge	1, 2,4
Cost-effectiveness	Hospital cost	Total hospital costs, taking into account the total length of hospital stay, operating room charges, medical and surgical supplies, pharmacy, laboratory and pathology, recovery room, anaesthesia, and ICU/observation rooms	1
	Overall cost	Cost of use of healthcare services; e.g. contact with a GP, in- or out-patient contact, prescribed medications	1
	Cost-effectiveness ratio	Cost-effectiveness of treatment route (medical management or surgery to remove the gallbladder), calculated by dividing cost by success rate (defined by the quality of life after	1

For peer review only - http://bmjopen.bmj.com/site/about/guidelines.xhtml

		treatment)		

1. Trials of interventions for gallstone disease
2. Patient reported outcome measures
3. Qualitative evidence synthesis
4. Primary qualitative research

For peer review only

For peer review only - http://bmjopen.bmj.com/site/about/guidelines.xhtml

Figure 1: PRISMA systematic review diagram for evidence synthesis. PRISMA= Preferred Reporting Items for Systematic Reviews and Meta-Analyses

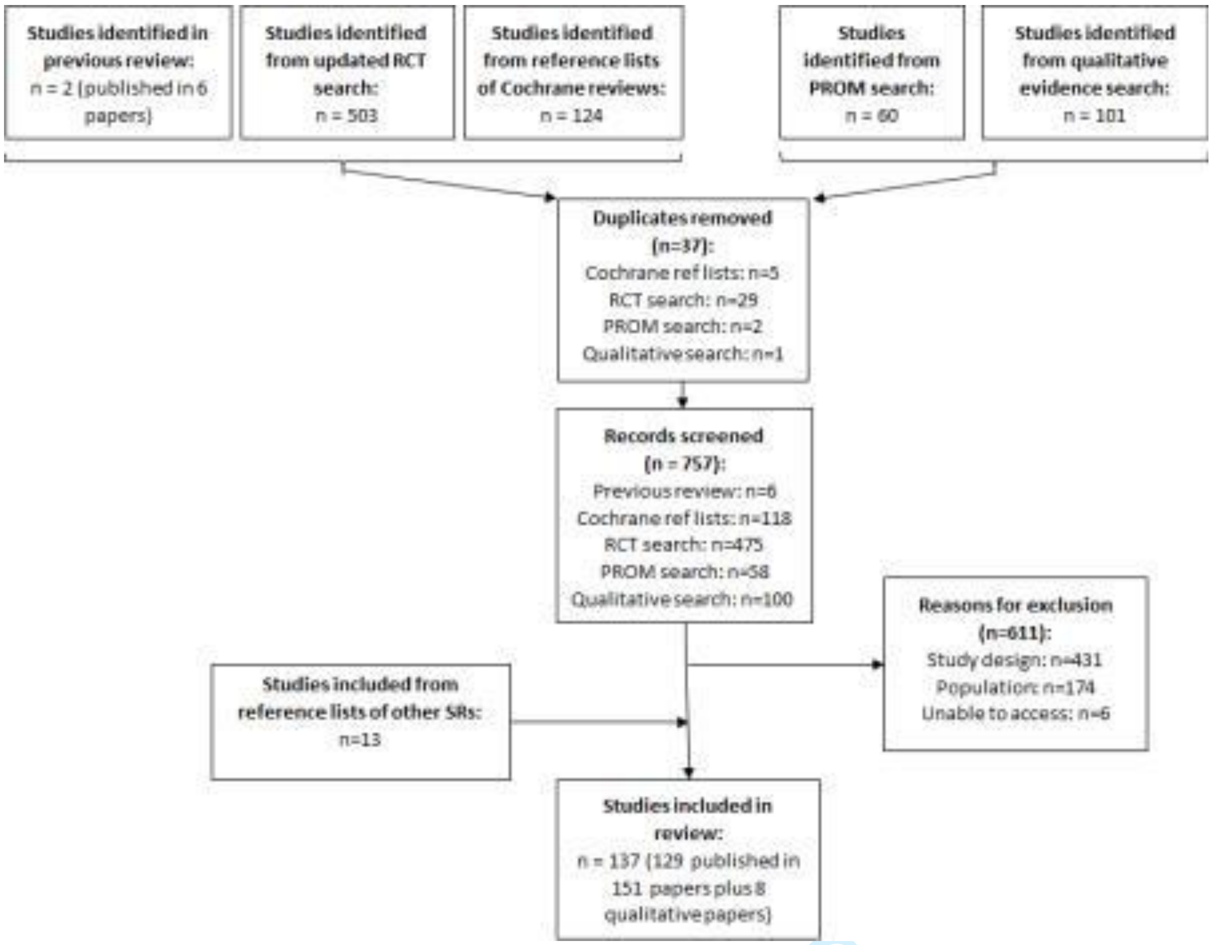

APPENDIX 1

UPDATED SEARCH STRATEGY TO IDENTIFY CLINICAL EFFECTIVENESS STUDIES

Ovid MEDLINE(R) Epub Ahead of Print, In-Process & Other Non-Indexed Citations, Ovid MEDLINE(R) Daily and Ovid MEDLINE(R) **1946 – 3rd May 2016** Embase **1980 to 2016 Week 18**

Date of Search: 3rd May 2016

1 cholecystitis/

2 cholecystitis, acute/

3 cholecystolithiasis/

4 gallstones/

5 cholelithiasis/

6 biliary colic/

7 (gall?bladder adj3 (empyema or inflam$)).tw.

8 (biliary colic or gall?stone$ or cholecystitis or cholecystolithiasis).tw.

9 ((pain or biliary symptom$) adj5 (cholecystitis or cholecystolithiasis or gall?bladder)).tw.

10 or/1-9

11 exp Cholecystectomy/

12 cholecystectom$.tw.

13 ((excis$ or remov$) adj4 gall?bladder).tw.

14 ((surgery or surgical) adj5 (cholecystitis or cholecystolithiasis or gall?bladder)).tw.

15 or/11-14

16 exp clinical trial/

17 randomized controlled trial.pt.

18 controlled clinical trial.pt

19 randomi?ed.ab.

20 randomly.ab.

21 trial.ab.

22 placebo.ab.

23 drug therapy.fs.

24 groups.ab.

25 comparative study/

26 (prospective$ or retrospective$).tw.

27 (compare$ or compara$).ti,ab.

28 or/16-27

29 10 and 15 and 28

30 (review or editorial or case report$ or letter).pt.

For peer review only

31 29 not 30

32 limit 31 to human

SEARCH STRATEGY TO IDENTIFY PROMS IN CHOLECYSTITIS

Ovid MEDLINE(R) Epub Ahead of Print, In-Process & Other Non-Indexed Citations, Ovid MEDLINE(R) Daily and Ovid MEDLINE(R) **1946 – 3rd May 2016** Embase **1980 to 2016 Week 18**

Date of Search: 3rd May 2016

1. exp cholecystitis/
2. cholecystolithiasis/
3. gallstones/
4. biliary colic/
5. (gall?bladder adj3 (empyema or inflam$)).tw,kw.
6. (biliary colic or gall?stone$ or cholecystitis or cholecystolithiasis).tw,kw.
7. ((pain or biliary symptom$) adj5 (cholecystitis or cholecystolithiasis or gall?bladder)).tw,kw.
8. or/1-7
9. (core adj3 outcome?).tw,kw.
10. (patient reported adj3 outcome?).tw,kw.
11. prom.tw,kw.
12. 8 and (9 or 10 or 11)
13. *outcome assessment/
14. *"Outcome Assessment (Health Care)"/
15. 8 and (13 or 14)
16. 12 or 15

SEARCH STRATEGY TO IDENTIFY QUALITATIVE STUDIES

Epub Ahead of Print, In-Process & Other Non-Indexed Citations, Ovid MEDLINE(R) Daily and Ovid MEDLINE(R) <1946 to 2nd May 2016

Date of Search: 18th of August, 2016

1. exp cholecystitis/
2. cholecystolithiasis/
3. gallstones/
4. exp Cholecystectomy/

For peer review only

5. biliary colic/

6. (gall?bladder adj3 (empyema or inflam$)).tw,kw.

7. (biliary colic or gall?stone$ or cholecystitis or cholecystolithiasis).tw,kw.

8. ((pain or biliary symptom$) adj5 (cholecystitis or cholecystolithiasis or gall?bladder)).tw,kw.

9. or/1-8

10. qualitative research/

11. exp interviews as topic/

12. focus groups/

13. grounded theory/

14. (qualitative or interview$ or focus group?).tw,kw.

15. (ethno$ or grounded or thematic or interpretive or narrative or discourse analysis or discursive or mixed method$).tw,kw.

16. or/10-15

17. 9 and 16

18. exp animals/ not human/

19. 17 not 18

For peer review only

Appendix 2 . Demographics of participants included in the primary research

**Research type and number of participants** (n)	**Gender**	**Approached to take part in the CGALL trial**<br>Yes/No and number (n)	**Trial consenters (allocated to receive surgery or medical management) or decliners**
Interviews<br>(n=6)	Female=6	Yes (n=6)	Allocated surgery=4<br>Allocated medical management=1<br>Trial non-consenter=1
Audio-consultations<br>(n=20)	Female= 16<br>Male=4	Yes (n=20)	Allocated surgery= 4<br>Allocated medical Management=3<br>Trial non-consenter=13
Focus Groups<br>(n=5)	Female=4<br>Male=1	No	Not Applicable

For peer review only
